# Dynamic Organization of Cells in Colonic Epithelium is Encoded by Five Biological Rules

**DOI:** 10.1111/boc.70017

**Published:** 2025-07-14

**Authors:** Bruce M. Boman, Thien‐Nam Dinh, Keith Decker, Brooks Emerick, Shirin R. Modarai, Lynn M. Opdenaker, Jeremy Z. Fields, Christopher Raymond, Gilberto Schleiniger

**Affiliations:** ^1^ Department of Mathematical Sciences Center for Applications of Mathematics in Medicine Newark Delaware USA; ^2^ Department of Computer & Information Sciences University of Delaware Newark Delaware USA; ^3^ Department of Mathematics Kutztown University of Pennsylvania Kutztown Pennsylvania USA; ^4^ Center for Translational Cancer Research Helen F. Graham Cancer Center & Research Institute Newark Delaware USA; ^5^ CATX Inc. Princeton New Jersey USA

## Abstract

Tissue organization is fundamental to the form and function of most, if not all, multicellular organisms. But what specifies the precise histologic organization of cells in tissues of plants and animals is unclear. We hypothesize that a *tissue code* exists that is the basis for dynamic maintenance of tissue organization. Any code for tissue organization must account for how the dynamics of tissue renewal maintain histologic structure. Accordingly, we modeled the dynamics of crypt renewal to determine how the organization of cells is maintained in colonic epithelium. Specifically, we modeled spatial and temporal asymmetries of cell division and established that five simple mathematical laws ([1] timing of cell division, [2] temporal order of cell division, [3] spatial direction of cell division, [4] number of cell divisions, and [5] cell lifespan) constitute a set of biological rules for colonic epithelia. Our results indicate that these rules form the basis of precise organization of cells in colonic epithelium during continuous crypt renewal. These five laws might even provide a means to understand the mechanisms that underlie organization of other tissue types, and how genetic alterations cause tissue disorganization that leads to birth defects and tissue pathology like cancer.

## Graphical abstract

This study reports that a set of five biological rules encodes how colonic epithelium dynamically maintains its precise organization of cells despite continuous tissue renewal. These rules might even provide a means to understand the mechanisms that underlie organization of other tissue types, and how tissue disorganization leads to birth defects and tissue pathology like cancer.

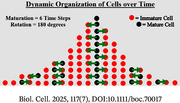

## 1 Introduction

Our *Ultimate Goal* is to determine if a set of rules (a tissue code) exists that explains the fidelity of tissue organization. For that, one needs to know how cellular mechanisms for tissue renewal encode the organization of cell populations in a tissue, that is, identify a code that explains tissue organization. A tissue code could provide a set of rules by which all multicellular organisms maintain themselves in a highly ordered state. Our immediate objective was to identify a set of mathematical rules that shows how human colonic epithelium is maintained during crypt renewal.

The critical *Gap‐in‐Our‐Knowledge* is how do multicellular organisms maintain organization of cells in their tissues? Scientists assume, it appears, that tissue organization is merely a process of cell proliferation that happens during tissue renewal. But that is insufficient to explain the precision of tissue renewal such as the constancy of tissue size, the subdivision of different tissue types (epithelia, connective tissue, muscle, and nervous tissue), the histologic pattern and unique distribution of specialized cells in each tissue, as well as the precise spatial relationships between individual cells. The tissue code could explain how multicellular tissues are normally organized, how that organization is faithfully maintained through tissue renewal over an organism's lifetime, and also how healing occurs after injury. Indeed, the existence of plant and animal life on earth for hundreds of million years necessitated an ability to precisely establish and maintain tissue organization.

Determining how the organization of tissues is encoded through tissue renewal presents a highly complex problem in biology. Historically, early efforts toward discovery of the genetic code held that the problem was vastly complex. It was mathematics that was instrumental in the initial discovery of the genetic code and how the information in DNA is transferred to proteins (Watson 1968; Crick et al. 1957). Taking this lesson from history, we used mathematical modeling to see if a set of rules could be found that explains how the complex organization of cells in tissues happens in healthy adults. In designing our model, we assumed that asymmetric division is key and must involve temporal as well as spatial mechanisms. An iterative approach was taken to create different models in the search of a model design that could simulate ongoing maintenance of cellular organization that happens during the continuous turnover of cell populations in a tissue. This involved repeatedly refining models through cycles of design, implementation, simulation, and evaluation which allowed for continuous improvement as new information emerged. This is standard approach in mathematical modeling of complex systems such as the emerging tissue organization in biology. After many iterations and refinements, we identified a set of mathematical laws that may explain how tissues could stay organized. We also then developed computer graphics to visually display the system dynamics. We developed both discrete and continuous models. Discrete models allowed us to observe the time evolution of individual cell patterns in tissue. Continuous models are better suited to obtain measures of tissue dynamics described by systems of differential equations.

Another reason that we turned to mathematics was that the mechanisms responsible for maintaining tissue organization were too complex to be easily solved using conventional biological experiments. That would require measurement of the dynamics of different cell types in tissues in vivo. Even recent exciting efforts to map whole organisms using image registration and gene expression analysis of all cell types in lower multicellular organisms (e.g., planarians) generate data on only a snapshot in time of the entire animal rather than continual dynamic motion of cells in various tissues (Fincher et al. 2018; Plass et al. 2018). Indeed, simultaneous recording of viable cells in terms of their dynamics (rate of cell movement; time of maturation and of division; the positions of cells relative to each other; direction of movement and of division) in living tissues would be necessary to understand the rules for tissue renewal. However, once these rules are identified mathematically, then biological experiments might be done to validate them.

In previous studies, models for morphogenesis were created to explain how embryonic tissues become organized during development. Indeed, such models have been designed based on mechanics (e.g., adhesion, forces), geometry (e.g., cell shape), reaction‐diffusion (pattern formation), and molecular mechanisms (involving genes, proteins, enzymes, and signaling pathways) (Maini 2006; Urdy 2012). And these models have provided valuable information as to how tissues are spatially formed in the early embryo during development. Some topological models have also been made that describe the geometric spatial organization of adult epithelia (Gibson and Gibson 2009). But they do not explain how adult tissue organization is dynamically maintained.

Investigating mechanisms in the dynamics of tissue renewal is a daunting challenge not only for the precision by which tissue organization is unerringly maintained, but also from the rapidity with which tissues replace themselves. In fact, healthy tissue organization in humans is preserved during the replacement of ∼2 trillion cells that turnover every day. This amounts to well over a million, million, million (>10^18^) cell divisions during the ∼60 years of average human adulthood. Given this extensive cell turnover, it is remarkable that the organization of any given tissue is continuously and precisely maintained in our organs. For example, we have been modeling the dynamic properties of tissue renewal of the human colonic epithelium, which takes 3–5 days (Boman et al. 2001; Boman et al. 2008). During self‐renewal in gut, the epithelium stays highly organized in terms of its specific cellular organization as well as distribution and number of crypts. Most other tissue types, even liver and brain, have their characteristic turnover time which continues throughout adulthood (Milo and Phillips 2015), as does the precise cellular organization of each tissue. The speed, precision, and complexity of tissue renewal processes strongly suggest the existence of an underlying *tissue code*.

To discern rules that define a tissue code, we reasoned that because tissue renewal is integrally linked to maintenance of adult tissue organization, the fidelity of tissue renewal must involve mechanisms that control cell division. We also surmised that any code that maintains tissue organization must contain biological rules that specify: (i) precise histologic structure, (ii) steady state dynamics, and (iii) tissue size. Hence, we conjectured that rules that determine histologic organization involve timing, temporal order, and direction of cell division. We also surmised that once a specific histologic pattern is established, other rules must underlie the steady state dynamics and size of the tissue. Thus, we conjectured that terminal differentiation (whole maturation), cell lifespan, and feedback mechanisms play a role in maintaining steady state dynamics and tissue size. Indeed, it was this line of reasoning that catalyzed our quest to investigate if maintenance of tissue organization can be explained by a set of simple rules.

Other sets of rules have been established (mainly reaction‐diffusion models) based on Alan Turing's ideas, that explain formation of tissue patterns in development. These models typically involve an activator and an inhibitor that diffuses based on an underlying random process (Turing 1952; Murray 2003; Kondo and Miura 2010). Because of the highly organized nature of tissues, in our model, the division and movement of cells were designed to be non‐random and to give rise to emergent behavior. The classic model that displays emergent behavior is Conway's game of life (Gardner 1970), but the patterns generated from Conway's model's output do not simulate the organization of tissues. In our view, cells within tissues possess the collective intelligence necessary for maintaining tissue organization because they inherit, from parental cells, instructions for timing (duration), temporal order (sequence), and spatial direction of cell division. So, our objective was to build a mathematical model of the tissue code in the form of a set of rules that was general enough and versatile enough that it can be applied to various types of tissue. By specifying settings (i.e., parameter values in our model) for the rules for each tissue type, our tissue code should at least qualitatively, and perhaps even quantitatively, be able to explain the organization of any given tissue type based on the code's many possible permutations.

Indeed, investigating how normal tissue organization is maintained with high fidelity is becoming one of the most significant questions in science today. For example, one of NSF's 10 big ideas is “Rules of Life”, which provides an agenda for “bold questions that will drive NSF's long‐term research” (Rules of Life [RoL]: Forecasting and Emergence in Living Systems [FELS]). To achieve NSF's goal to “understand the organizational principles and rules of living systems”, scientists will be seeking answers to questions such as how tissues faithfully sustain highly ordered structures, like our quest to seek a code for tissue organization.

## 2 Methods

Consequently, we created two mathematical models (one discrete, the other continuous) that account for healthy adult tissue's main properties: (i) cells are the constituent parts or building blocks that make up tissues, (ii) tissues continuously turnover, (iii) the number of cells in tissues is maintained constant, and (iv) the organization of cell types within tissues is preserved during tissue renewal.

To account for such properties, we designed our model based on temporal asymmetry of cell division as proposed by Spears and Bicknell‐Johnson (1998). In this design, division of a mature parent cell produces two progeny cells, a mature (M) cell and an immature (I) cell, with different temporal properties. An earlier version of our model (Boman et al. 2017), which built upon this temporal asymmetry, simulated dynamics of tissue renewal, but it did not explain how adult tissue organization is maintained. Unlike the previous model (Boman et al. 2017), our current model accounts for cell rotation of cell division. Accordingly, we incorporated new mechanisms for timing, temporal order, and spatial direction of cell division (*Rules* 1–5, see 3.1 below). Our discrete model accounts for each cell in the system with rules for spatial and temporal instructions for cell division. Our continuous linear model assumes a large number of cells, and accounts for the relative proportion of each cell type. The continuous model was created to provide quantitative measures of discrete model system dynamics.

### 2.1 Asymmetric Division and Rotation

In our basic discrete model, the daughter cell inherits the split‐angle of its parent cell (Figure S1, Supporting File 1), which turns counterclockwise by one increment every time step. Given this process, leaflets of I cells form around the M cell. Model output eventually creates branches that emerge from the leaflets (also see Supporting File 1).

### 2.2 Agent‐Based Model

An agent‐based model was formulated that incorporates *Rules 2–*5 with the agents being the cells. Maturation (*c*), rotation (*R*), whole‐maturation time (*n*_wm_), and lifespan (*L*) specify the age at which cells change state. At age zero, a cell is immature; at age *c*, a cell becomes mature; at age *n*_wm_, a cell becomes wholly‐mature; at age *L*, the cell reaches the end of its life cycle and dies. The number of divisions undergone by any given cell is determined by: #divisions = *n*_wm_ − *c*. The clonogenic cell remains immortal because it defines generation zero and continually generates I cells. Thus, its *c* value defines the pattern of the entire structure. The computer code is available upon request.

### 2.3 Modeling Steady‐State Tissue Renewal

To find settings that establish steady‐state tissue renewal *n_wm_* values were adjusted while keeping the *c* value constant such that every M cell under a steady‐state condition only produces, on average, a single I cell. Namely, *n*_wm_ was programmed to decrease over time as a function of cell generation (*g*) whereby *n*_wm_ = *n*_wm,0_−*g*, where *n*_wm,0_ is an initial constant value. Cell generation is the number of divisions removed from the zeroth generation clonogenic cell. Since the number of divisions of a cell is defined as *n*_wm_−*c*, it is clear that when holding *c* constant, *n*_wm_ must be reduced for later generations to reach steady state. Thus, when *n*_wm_ is reduced as a function of *g*, different population sizes will be produced depending on the initial value of *n*_wm_ (*n*_wm,0_).

### 2.4 Continuous Model

In our continuous model (Figures S2 and S3, Supporting File 1) we created a simple, linear system of differential equations modeling the dynamics of 4 cell types (M, I, W_1_, W_2_).

### 2.5 Analysis of Human Colonic Crypts

Colonic crypts were purified from normal human colonic epithelium from surgical specimens as we previously described (Zhang et al. 2010; Huang et al. 2009; Zhang et al. 2020; Modarai et al. 2018). Isolated viable colonic crypts were analyzed for the ALDH stem cell marker by ALDEFLUOR analysis and paraformaldehyde fixed colonic crypts were analyzed for ALDH by immunofluorescence as we previously described (Zhang et al. 2010; Huang et al. 2009; Zhang et al. 2020; Modarai et al. 2018). Briefly we used anti‐ALDH1 (BD Pharmingen, Franklin Lakes, 1:50) as the primary antibody. The use of human tissues was approved by Institutional Review Board of Christiana Care Health Services, Inc (FWA00006557).

## 3 Results

### 3.1 Discrete Model

In creating the discrete model, through extensive iterative modeling, we were able to find one model design to simulate tissue organization during the continuous turnover of cell populations. In this design, the maturation period (*c* value) is linked to the degree of rotation (i.e., direction) of cell division, which mimicked the expected emergent behavior of cells in tissues. For this model design, the five rules we postulated for tissue organization were as follows:

Rule 1 Timing of cell division. The timing of cell division is based on a fixed cell cycle duration. The timing of cell division of the two different proliferative cell types, mature (M) cells and immature (I) cells, is modeled on the duration of this cell cycle.

Rule 2 Temporal order of cell division. Asymmetric cell division of a mature (M) cell generates two cells, the parent M cell and an immature (I) progeny cell, which have different temporal *dynamic* properties. Specifically, the temporal order of division of these two cells (i.e., the sequence in which cell divisions occur over time) is that M cells divide every cell cycle and I cells divide only after a maturation period (*c* value). Once an I cell undergoes maturation, it immediately becomes an M cell and it divides (i.e., undergoing cell division is the condition for maturation).

Rule 3 Spatial direction of cell division. The direction of cell division rotates by a fixed angle, every cycle, which is a function of the maturation period (c value). During cell division, each M and I daughter cell inherits the instructions for the direction and timing of its next cell division.

Rule 4 Number of cell divisions (n). The period during which M cells can divide to generate I cells is limited by the whole maturation time n_*wm*_, or age when an M cell becomes wholly mature, that is, terminally‐differentiated, which is a function of generation number g of a cell, namely, the number of divisions a stem cell has undergone to produce the given cell. The age of a cell is the number of time steps n, that is, the number of divisions since the cell was produced. When an M cell reaches this limit, it can no longer proliferate, and it becomes a wholly mature (W) cell. Immature (I) cells also become wholly mature if maturation period equals whole maturation time (c = n_*wm*_). This rule dictates the number of divisions that a cell will undergo.

Rule 5 Cell lifespan. The lifespan of a cell (L) is the time that it exists in the tissue for the period from birth to death.

Thus, the behavior of each cell is defined by three immutable properties: a maturation‐cycle, a time to reach whole‐maturation, and a lifespan, which are abbreviated as *mc, wm, and L*, respectively. Together, these internal properties comprehensively govern the evolution of a cell agent and are summarized in Table 1.

**TABLE 1:** Internal properties tracked by cells.

Property	Description
Age	Number of time steps since division
Generation	Divisions removed from stem cell
Split angle	Angle of the next division
Maturation cycle (*mc*)	Age at maturation
Whole‐maturation time (*wm*)	Age at whole‐maturation
Lifespan (*L*)	Age at death

*Note*: Of these generation, *mc, wm*, and *ls* remain constant during lifetime of a cell while age and split angle are continually incremented.

Asymmetric division (*Rule* 2) is modeled as a simple splitting mechanism. Figure 1A shows a cell lineage diagram that illustrates how asymmetric cell division generates an M cell and an I cell, and that I cells divide only after a given maturation period (*c* value). Even in this simple linear case, it is seen that a dynamic self‐renewing cell pattern emerges. This same model output can be plotted in other ways to illustrate how the rules for asymmetric cell division affect tissue organization. For example, Figure 1B displays the pattern of cells that are produced according to cell generation (parent, 1st, and 2nd). Figure 1C,D also show the pattern of cell production based on a tree diagram. The numbered tree diagram (Figure 1D) tracks the sequence of division, age, position, and number of cells over time. The order of cell divisions up to time step 15 is also given in Table 2. The order of cell divisions gives rise to the number of cells in each different time cycle that fits the Fibonacci *p*‐number sequence (A005708). In all three illustrations (Figure 1A–C), it is observed that the model output produces a dynamic pattern of a self‐renewing system while the organization of cells in the tissue is maintained constant.

FIGURE 1Discrete model for asymmetric cell division. (A) Displays the production of I and M cells (*Rule 2*) over time for maturation period *c* = 6 based on a simple linear model. Time periods are from top to bottom. Production of cells occurs successively from left to right (new cells displace old cells to the right). It illustrates: (i) The process of asymmetric cell division (division of an M cell that produces an M cell and I cell); (ii) The time (six cycles) it takes for I cells to mature into M cells and divide; and (iii) The formation of a pattern of cellular organization created by division of M cells within the overall population. The first new cell produced in the first‐time cycle is marked by an asterisk (*) to show its location as the system progresses in size over time. Notably, even though all M cells in the population divide every cycle, the cellular organization of the population stays exactly the same. (B) Displays the pattern of cells that are produced across different generations (parent, 1st, and 2nd). M cells are black and I cells are red. The parent (zero) generation cell is the clonogenic cell, which is a M cell that asymmetrically divides every time cycle to produce an I cell. At time cycle 7, the oldest 1st generation I cell matures into an M cell and divides to produce a 2nd generation I cell. At time cycle 12, there are twelve 1st generation cells and twenty‐one 2nd generation cells. (C) Shows a tree diagram of cell production based on maturation period *c* = 6 that occurs over 12‐time cycles. In the upper tree display, the clonogenic cell is white, I cells are red, and M cells are blue. The clonogenic cell divides every time cycle to produce daughter cells that will give rise to the whole of the tree structure. Note that the clonogenic cell divides asymmetrically from right to left and M cells divide asymmetrically from left to right. This plot of division of the clonogenic cell and M cells in opposite directions shows that the oldest 1st generation cell always moves furthest away (to the left) from the clonogenic cell. (D) This numbered tree design corresponds to the tree diagram in (C). The cells are numbered according to cell division that occurs asymmetrically to generate an older daughter and younger daughter cell. Accordingly, each cell has an age label that is numbered according to the sequence of cell division. Once a cell is numbered, it always keeps that age label. The order of cell divisions gives rise to the number of cells in each different time cycle that fits the Fibonacci *p*‐number sequence (A005708). The figure gives output up to time step 12. After time step 12, the clonogenic cell (#0) continues to divide asymmetrically from right to left and all other cell divisions divide asymmetrically from left to right. For example, in time step 13, cells #7 and #8 will become mature cells and divide to produce daughter cells #41 and #42, respectively. The younger daughters #41 and #42 will be positioned to the right of older cells #7 and #8. The order of cell divisions up to time step 15 is given in Table 2. (E) shows the incorporation of rotation (by 60 degrees) into our model of asymmetric cell division. In other words, instead of I cells being produced in a linear fashion from M cells in (A–D), (E) shows that I cells are produced counterclockwise around the M cell. This displays the pattern produced by modeling according to a simple splitting mechanism (*c* = 6) where cells divide in 60‐degree increments determined by line (m) running through the cell center and perpendicular to the M cell (black circle) splitting orientation (solid black arrow). During cell division, both the I cell (red circle) and the M cell: (i) inherit a splitting orientation that is the same as the original direction and (ii) rotate by angle *R* counterclockwise about their current orientation (*Rule* 3). The M cell then becomes the grid center and the I cell holds its position relative to the center of the grid. The process repeats itself with the M cell and its new “m” line. When an I cell is produced in an occupied space, the older I cell becomes displaced one position away and in the appropriate direction. Thus, at time cycle 7, the oldest 1st generation I cell matures into an M cell is displaced one position away and divides to produce a new 2nd generation I cell (see lowest cell pattern in (E)). In this way, even though the population of cells is growing, the organization of I and M cells in the population stays exactly the same. Continuation of the process generates an organized pattern of leaflets and branches that simulates tissue rosette structures in histology.

**Figure:**
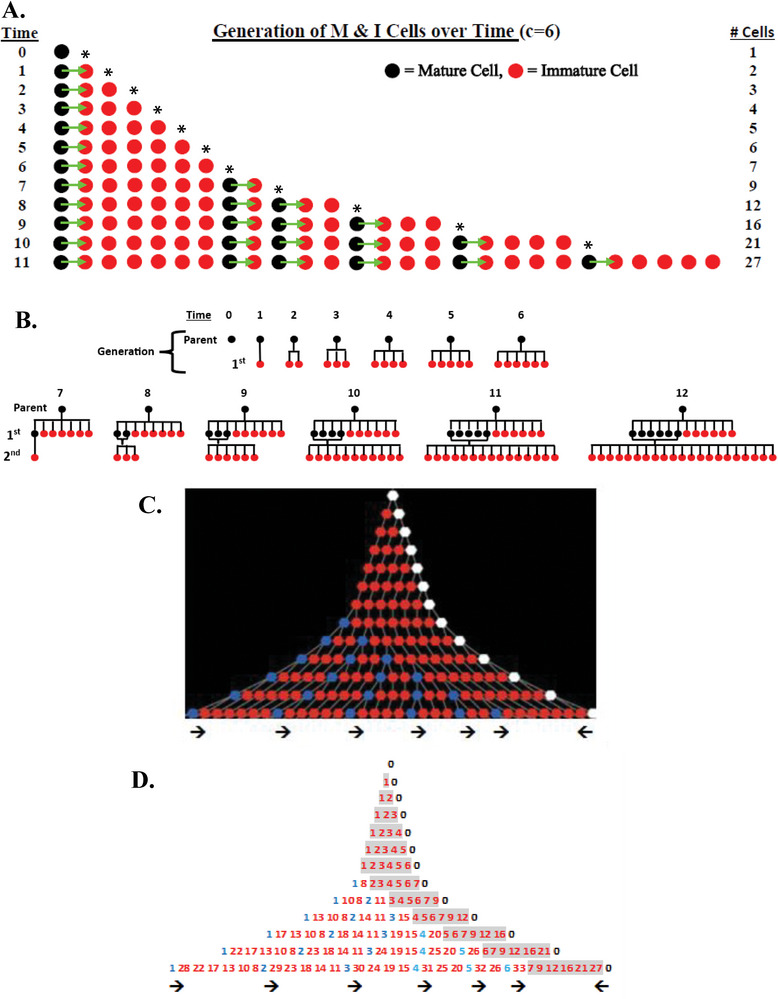

**Figure:**
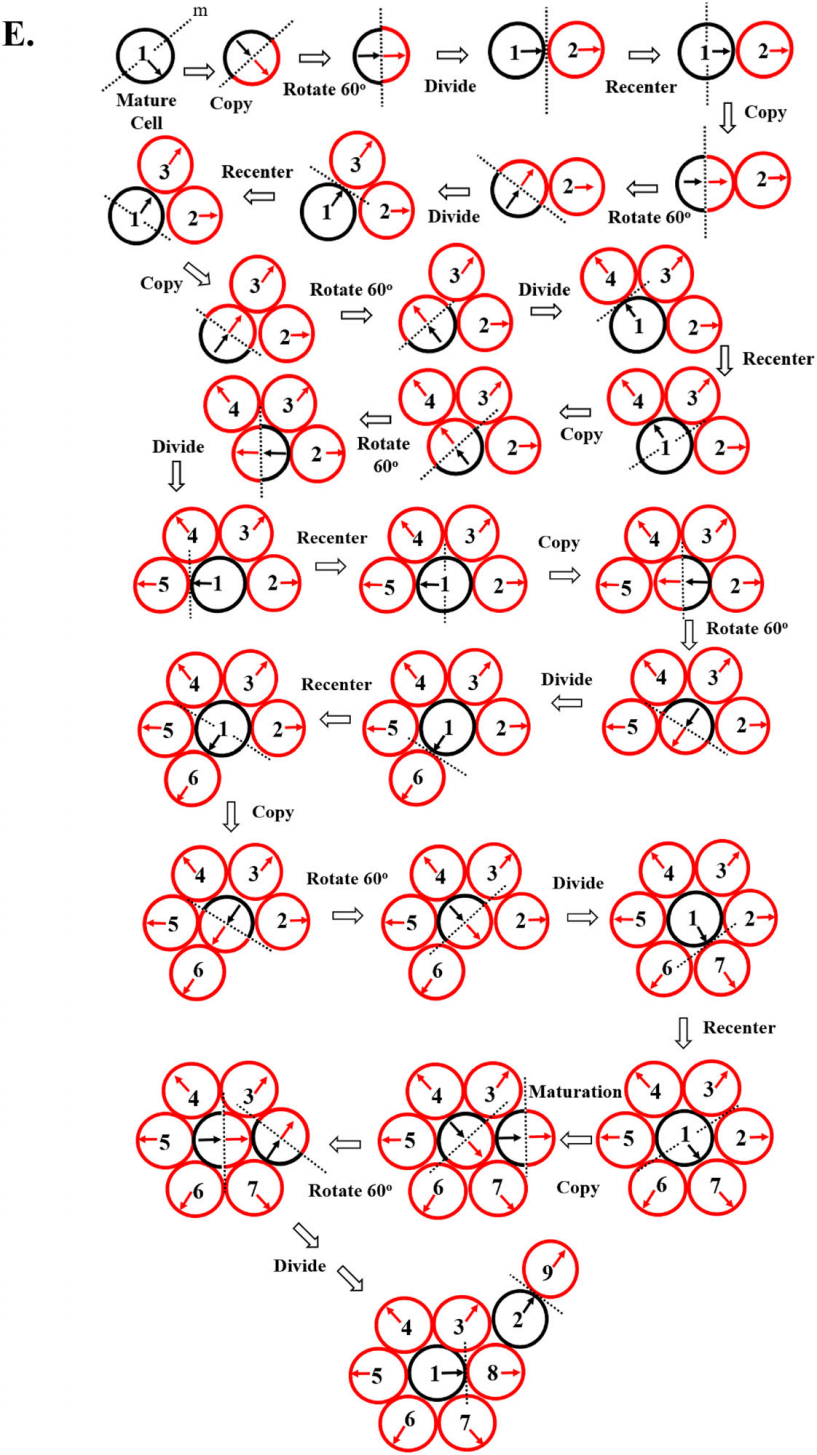

**TABLE 2:** Order of cell divisions.

Time	Order of cell divisions	#Cells
0	0	1
1	0→1	2
2	0→2	3
3	0→3	4
4	0→4	5
5	0→5	6
6	0→6	7
7	0→7 1→8	9
8	0→9 1→10 2→11	12
9	0→12 1→13 2→14 3→15	16
10	0→16 1→17 2→18 3→19 4→20	21
11	0→21 1→22 2→23 3→24 4→25 5→26	27
12	0→27 1→28 2→29 3→30 4→31 5→32 6→33	34
13	0→34 1→35 2→36 3→37 4→38 5→39 6→40 7→41 8→42	43
14	0→43 1→44 2→45 3→46 4→47 5→48 6→49 7→50 8→51 9→52 10→53 11→54	55
15	0→55 1→56 2→57 3→58 4→59 5→50 6→61 7→62 8→63 9→64 10→65 11→66 12→67 13→68 14→69 15→70	71

*Note*: The table gives the order of cell divisions (for *c* = 6) up to time step 15. This table corresponds to Figure 1D. The cells are numbered according to cell division that occurs asymmetrically to generate an older daughter and younger daughter cell. Accordingly, each cell has an age label that is numbered according to the sequence of cell division. Once a cell is numbered, it always keeps that age label. The order of cell divisions gives rise to the number of cells in each different time cycle that corresponds to the Fibonacci *p*‐number sequence (A005708)

When rotation of cell division (*Rule* 3) is integrated into this splitting mechanism, more complex self‐renewing patterns are generated. In this situation, the *c* value defines the direction that an M cell rotates (degree *R*) during asymmetric division, and the number of leaflets is based on *R*. Figure 1E shows how cellular organization emerges based on the link between maturation (*c* = 6) and rotation (60 degrees). For other settings of *Rule*s 2 and 3, various patterns can generated that demonstrate a specific organization of cells, leaflets, and branches (see Figure S1, Table S1, and agent‐based simulation below).

**FIGURE 2:**
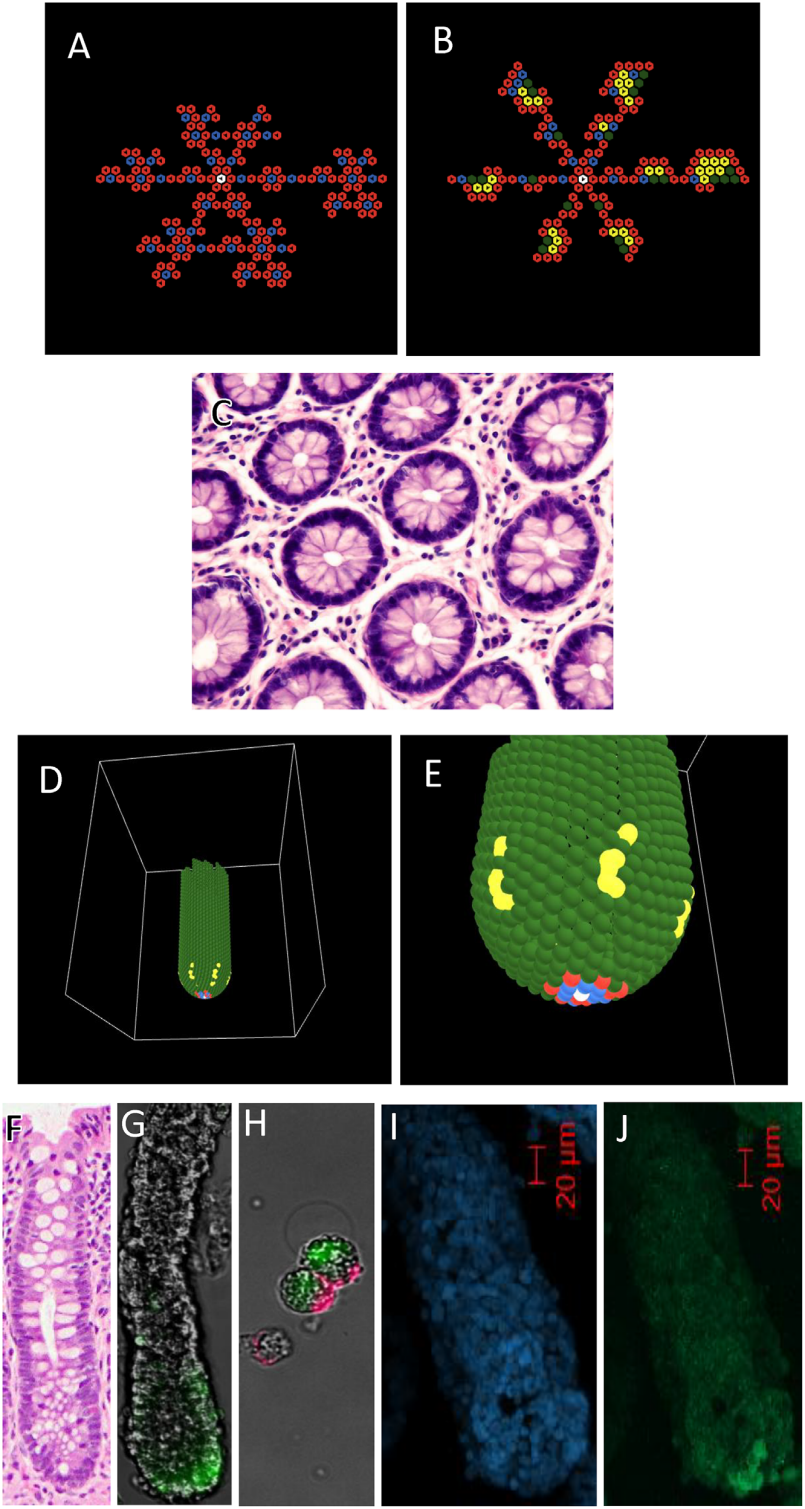
Model output. For any chosen *c* value in these runs, the computer code output produces an expanding geometric structure defined by the active division of M cells located within the growing branches that surround the clonogenic cell. (A, B) show 2D output for an agent‐based model run based on a maturation time of *c* = 6. (A) Shows a snapshot in time of 2D model output (*n*_wm_ = ∞; *L* = ∞ up to time step 24). (B) Shows an example of a steady state structure implemented with *c* = 6; *L* = 35, *n*_wm_,_0_ = 11 and *n*_wm_ decreased linearly with *g* (*n*_wm_ = *n*_wm,0_ − *g*). Here, *L* is set large enough to ensure sufficient time for development of the model's structure while still maintaining steady‐state. (B) Gives a cell‐level view up to time step 27. Note the cells eventually die near the edges of the structure. The clonogenic cell is colored white, I cells red, M cells blue, wholly‐mature cells green, and overlapping cells yellow. Arrows indicate next split‐direction. (C) is the image of an H&E‐stained tissue cross‐section of normal colonic epithelium showing the highly organized structure of colonic crypts in human tissue. Note that the colonic epithelium is a single cell sheet of interconnected colonocytes. Throughout life, this organization is precisely maintained during colonic crypt renewal that replaces the epithelium with a turnover rate of 5 days. Our 2D model output simulates how this dynamic organization might be maintained during colonic tissue renewal. (D, E) Show a steady state structure in 3D that simulates organization and dynamics of human colonic crypts (single cell thickness wall with 42 cells columnar circumference). In this perspective, the 3D structure in Figure 2D,E represents a vertical projection of the 2D structure in Figure 2A,B. The distribution of various model cell populations (clonogenic, immature, dividing‐mature, overlapping, wholly‐mature cells) simulates distribution of crypt cell types (stem, progenitor, transit‐amplifying, phagocytized apoptotic, terminally‐differentiated cells) in biology (Boman et al. 2001; Boman et al. 2008; Boman and Fields 2013; Zhang et al. 2010; Huang et al. 2009; Sträter et al. 1995; Potten 1992; Potten 1998). (F–J) Show the anatomic location of stem (clonogenic) cells at the bottom of normal human colonic crypts. The location of stem cells in crypts isolated from colonic epithelium was analyzed for the stem cell marker aldehyde dehydrogenase (ALDH) using the ALDEFLUOR assay for ALDH enzymatic activity (Huang et al. 2009; Zhang et al. 2020; Modarai et al. 2018; Facey et al. 2024). (F) is the image of an H&E‐stained longitudinal tissue‐section of a normal colonic crypt showing its highly organized structure. (G) Shows that ALDH‐positive stem cells (green stain) are located at the crypt bottom of viable colonic crypts. (H) Reveals an image of live cells from dissociated ALDEFLUOR‐stained colonic crypts that were co‐stained for the EpCAM epithelial marker (red) to validate that ALDEFLUOR (green) is staining crypt cells not stromal cells. The location of stem cells was also studied using paraformaldehyde‐fixed whole colonic crypts. (I) Shows a fixed crypt that was stained with DAPI for cell nuclei. (J) Shows the same fixed crypt immune‐stained with anti‐ALDH1 antibody revealing that ALDH+ cells reside at the crypt bottom. Thus, model output mimics dynamics of crypt renewal because cells are constantly dividing in regions of active proliferation in the lower crypt while the location and organization of all cell populations and structure (shape, size) stays the same despite a continuous stream of cell populations upward.

Our discrete model was also formulated as an agent‐based model. The initial simulations were done based on settings with no differentiation of cells to wholly mature cells (*n*_wm_ = ∞) and cells were immortal (*L* = ∞). For any chosen *c* value in these runs, the Net Logo code output produces an expanding geometric structure defined by the active division of M cells located within the growing branches that surrounded a clonogenic cell (Figure 2). Although the system in Figure 2A does not reach steady‐state, the run shows how the model generates symmetric patterns that have a specific organization of cells within branches, and where the organization of cells within the branches remains constant even though the cells within branches are continuously dividing and branches themselves are continually self‐renewing and expanding. The two‐dimensional structure generated from our agent‐based model simulates a tissue rosette.

To more closely simulate the dynamic organization of healthy tissues, we then sought to find model settings that establish steady‐state tissue renewal. This was done by adjusting *n*_wm_ values while keeping the *c* value constant. An example of a two‐dimensional steady state structure generated is shown in Figure 2B. In establishing a steady state, for any choice of the initial *n*_wm_, a region of active division is generated around the clonogenic cell and expanding branches constantly push out a stream of infertile W cells. Figure 2B shows model output for time steps of the process in which *n*_wm_ is reduced as a function of *g*. Although cell lifespan (*L*) does not play a part in the size of the cell production region, it limits the overall size of the structure at steady state conditions. Notably, the organization of cells displayed in two dimensions (2D) is maintained even though the cells within the structure are continuously being replaced. This output simulates the maintenance of tissue organization during tissue renewal in biology such as seen in the organization of crypts of Lieberkühn found in the large intestine (Figure 2C). Biologically, the organization of colonic epithelium happens through stem cell‐based processes involving both crypt renewal and crypt fission (Boman et al. 2001; Boman et al. 2008; Lopez‐Garcia et al. 2010; Snippert et al. 2010; Tomlinson and Bodmer 1995; Johnston et al. 2007). In our modeling, when the steady state structure is projected in three‐dimensional space (Figure 2D,E), it simulates the organization and dynamics of the human colonic crypt (Figure 2F–J). Since colonic crypts are comprised of a single sheet of cells that is configured as a tube‐like crypt gland (Figure 2F), the 3D structure (Figure 2D,E) can be modeled as a vertical projection of the planar 2D structure (Figure 2A,B). This fits with biology because long‐term lineage tracing studies in mice show the colonic crypt in made of distinct populations that form streams of cells that migrate from the stem cell niche at the bottom to the crypt top (Lopez‐Garcia et al. 2010; Snippert et al. 2010).

Our goal to simulate the organization of colonic crypt and the presentation of biological data here was done as a means to show that model output can mimic biological tissue renewal and organization. Although a number of important studies on modeling of colonic crypt kinetics have been published (Tomlinson and Bodmer 1995; Johnston et al. 2007; van Leeuwen et al. 2006), including our own studies (Boman et al. 2001; Boman et al. 2008), most of them do not display emergent cell behavior that simulates the dynamic continuous maintenance of organization of cells in colonic crypts during tissue renewal.

**FIGURE 3:**
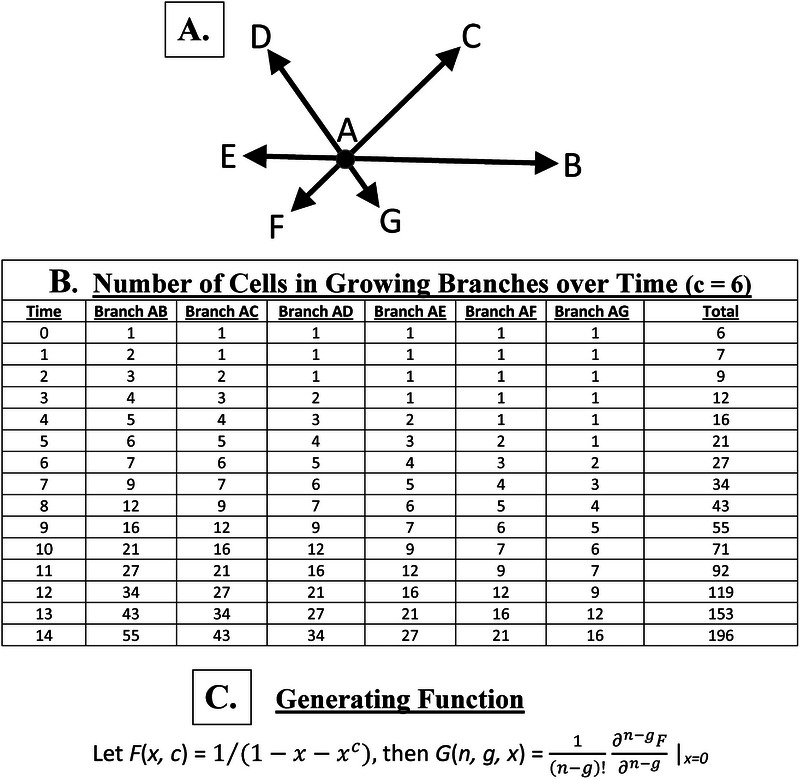
Model design. Our model is designed to produce an expanding geometric structure defined by the active division of M cells located within the growing branches that surround the clonogenic cell (as shown in Figure 3). (A) shows direction of expansion (arrows) of the six branches for a maturation time of *c* = 6. (B) shows output on the number of cells generated over time that corresponds to the six branches (A) which is computed based on the generating function (C), which gives the number of cells produced in different branches over time for any *c* value. Note that the number of cells for each branch does not include the clonogenic cell or the six cells in the first circumferential layer of leaflet cells surrounding the clonogenic cell. Also note that the number of cells generated over time fits the Fibonacci *p*‐number sequence for *p* = 6.

To compute the number of cells that are produced in the different branches as a function of time, we developed a generating function (Figure 3). Output on the number of cells generated over time for *c* = 6 that corresponds to the six branches (Figure 3A) is computed based on this generating function (Figure 3B,C). The numbers generated in growing branches for other *c* values (*c* = 2–5) are given in Tables S2 and S3. Model output shows that the total number of cells generated over time (for *n*_wm_ and *L* = ∞) based on any *c* value follows the generalized Fibonacci sequence:

$$F_{n}=F_{n-1}+F_{n-c}\;\mathrm{where}\;F_{0},F_{1},\text{…}F_{c-1}=1$$

### 3.2 Continuous Model

**FIGURE 4:**
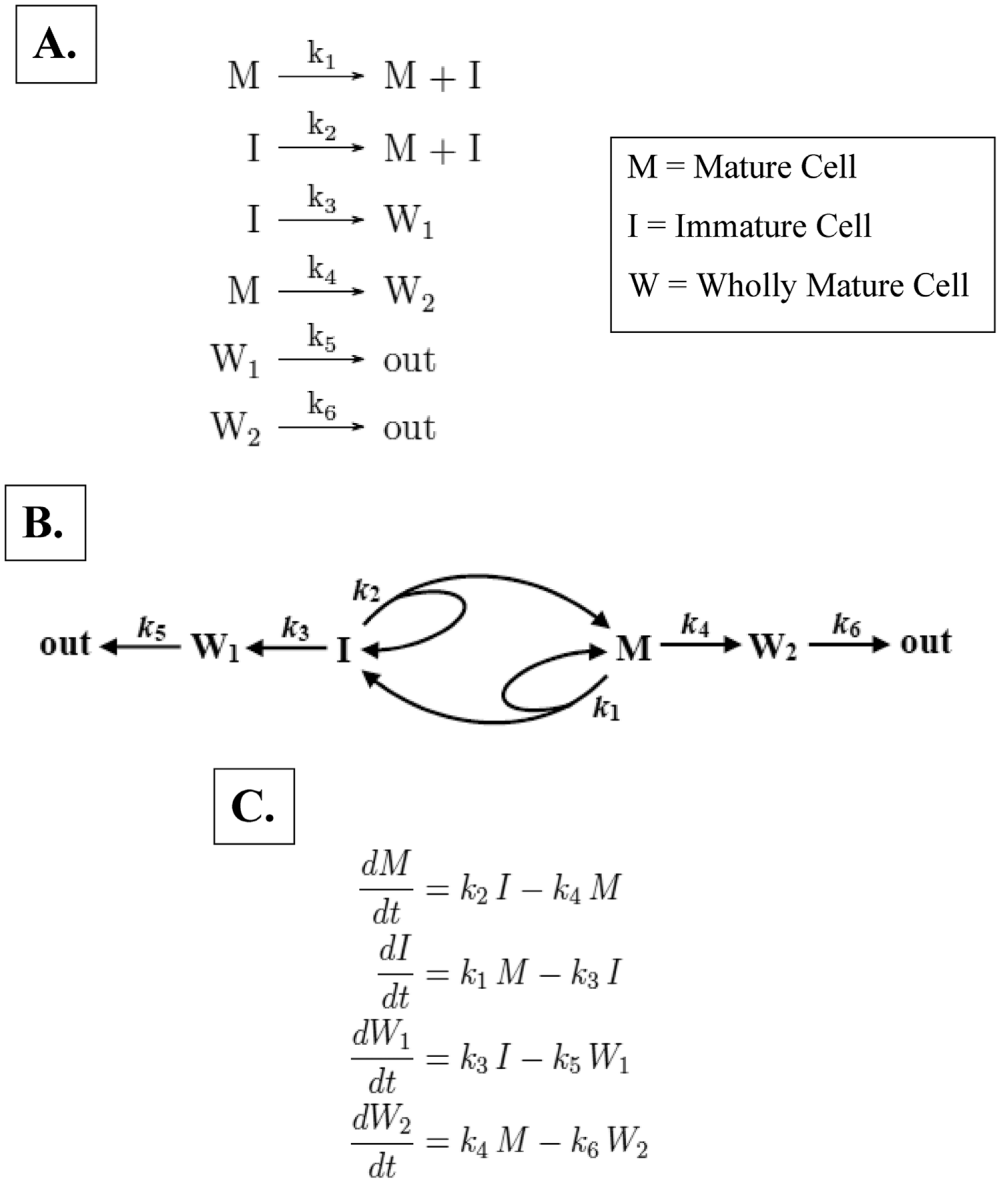
Continuous model for tissue renewal based on asymmetric cell division. Our continuous model has several features that simulate dynamic properties of cells in tissues. The model design is shown in (A,B), and rate equations and reaction rates in (C). The model shows that maturation of I cells and M cells involves their proliferation and differentiation, which occur simultaneously in cells of both populations. Self‐renewal occurs on two scales: (i) Individual I and M cells self‐renew (governed by *k*_1_ and *k*_2_ rate constants); (ii) The system itself self‐renews. For example, if M cells are depleted, they can be regenerated by division of I cells. Conversely, if I cells are depleted, they can be replenished by division of M cells. In other words, there is interconversion between the proliferative cell populations (i.e., between I and M cells), which provides a mechanism for cellular regeneration and, consequently, tissue renewal and even healing after injury. This model design fits with the biology of many tissues, including intestine, lung, stomach and skin, in which proliferative cell populations are known to self‐renew as well as to interconvert (Takeda et al. 2011; Rompolas et al. 2013; Stange et al. 2013; Tata et al. 2013). Asymmetric cell division results in two daughter cells that have different properties. One property is that the two daughter cells exist in different states of maturation which affects their rate of cell division—M cells divide every cycle (based on *k*_1_) whereas I cells divide at a slower rate (based on *k*_2_). An additional property is that the two daughter cells differentiate into different cellular lineages—I cells differentiate into W_1_ cells (based on *k*_3_) and, conversely, M cells differentiate into W_2_ cells (based on *k*_4_). Moreover, based on the model design, it is apparent that different steady states can be achieved in which the proportions of various cell types can differ from one steady state to the next. The achievement of a steady state requires a balance between the production and loss of proliferative cells from the division of M and I cells (governed by *k*_1_ and *k*_2_) and the whole maturation of W_1_ and W_2_ cells (governed by *k*_3_ and *k*_4_), respectively. Our modeling also shows, based on eigenvalue solutions, that differentiation along only one lineage will not achieve a steady state—it will only result in exponential growth or decay of the system (data not shown). Thus, differentiation along two lineages in our model is required to achieve a steady state. This result provides an explanation for why many tissues possess two main lineages for differentiation. Examples of such dual lineages in tissues includes hematopoietic (lymphoid and myeloid) (Görgens et al. 2013), brain (neuronal and glial) (Gage and Temple 2013), and intestine (secretory and absorptive) (Gracz and Magness 2014) types. Thus, our model design incorporates temporal and spatial properties of cells in tissues including asymmetric division, self‐renewal, interconversion, maturation, and steady state kinetics.

In addition to our discrete model, we created a continuous model (Figure 4A,B) to provide measures of discrete model system dynamics. The rate equations and reaction rates are given in Figure 4C. For the rate constant *k*_1_ for division of M cells, we can choose *k*_1_ = 1 without loss of generality. This just means that time will be measured in units of 1/*k*_1_ and the other rate constants will therefore be automatically scaled by *k*_1_. The rate constant value (*k*_2_) for division of I cells depends on the *c* value. The W_1_ and W_2_ cells in the continuous model correspond to W cells in the discrete model. The value of *k*_2_ can be determined by the long‐term output on numbers of M and I cells resulting from the discrete model (for each *c* value). The general solution to the system is given in Figure S2, which also provides expressions for the eigenvalues (λ_1,2,3,4_) as follows:

$$\begin{array}{ccc} \lambda_{1,2} & = & \frac{-\left(k_{3}+k_{4}\right)\pm \sqrt{{\left(k_{3}+k_{4}\right)}^{2}4\left(k_{3}k_{4}-k_{1}k_{2}\right)}}{2},\\ & & \lambda_{3}=-k_{5},\;\lambda_{4}=-k_{6}. \end{array}$$

Hence, three eigenvalues are negative (λ_2_ < 0, λ_3_ < 0, and λ_4_ < 0) and the other one, λ_1_ (λ_1_ > λ_2_), is zero, positive or negative depending on whether *k*_3_ *k*_4_ = *k*_1_ *k*_2_, *k*_3_ *k*_4_ < *k*_1_ *k*_2_ or *k*_3_ *k*_4_ > *k*_1_ *k*_2_, respectively. Thus, if *k*_3_ *k*_4_ = *k*_1_ *k*_2_, then the relative ratios at steady‐state are:

$$\frac{M(t)}{I(t)}=\frac{k_{2}}{k_{4}}=k_{3},\;\frac{W_{2}\left(t\right)}{M(t)}=\frac{k_{4}}{k_{6}},\;\frac{W_{1}\left(t\right)}{I(t)}=\frac{k_{3}}{k_{5}}.$$

We then studied how the continuous model might provide measures of discrete model system dynamics, particularly the behavior of cells in the region of active cell division around the clonogenic cell in the discrete model. We did this by comparing the discrete model data with continuous model output when parameter settings in both models produced exponential growth by excluding differentiation of M to W and I to W cells (i.e., *n*_wm_ = ∞; *L* = ∞ in discrete model; *k*_3_ = *k*_4_ = 0 in continuous model). In this case, for the continuous model, we find that λ_1_ = $\sqrt{k_{2}}$= $\frac{M}{I}$ (Table 3). For the discrete model, we find that $\frac{M}{I}$= ${\left(\frac{I}{M+I}\right)}^{c-1}$. Solving for $\sqrt{k_{2}}$ and *c*, the resultant expressions $\sqrt{k_{2}}$ ${\left(\sqrt{k_{2}}+1\right)}^{c-1}$−1 = 0 and *c* = 1−$\frac{ln\sqrt{k_{2}}}{ln(\sqrt{k_{2}}+1)}$ provide a correlation of $\sqrt{k_{2}}$ in the continuous model with the *c* value (nondimensional) in the discrete model.

**TABLE 3:** Relationship between rate constant values (k_1_, k_2_), eigenvalue (λ_1_), and ratio of mature (M) to immature (I) cells for different *c* values.

*c* value	*k*_1_	*k*_2_	λ_1_ (=$\sqrt{k_{2}}$)	M/I
1	1	1	1	1
2	1	0.381966	0.618034	0.618034
3	1	0.216757	0.465571	0.465571
4	1	0.144611	0.380278	0.380278
5	1	0.105442	0.324718	0.324718
6	1	0.081338	0.285199	0.285199

Comparison of model results also revealed that other correlations exist between the two models (Table 3). For example, discrete model output expressed as a quotient of the sizes of the growing branches for *c* = 2 to *c* = 6 can also be expressed as functions of *k*_2_ rate constant values from the continuous model (Figure S3).

Furthermore, the square roots of the rate constant values for different *c* values relate to the Fibonacci *p*‐numbers and golden *p*‐sections (Stakhov and Olsen 2009), which indicates that the rate equations and rate constants provide a means to explain growth of the *p*‐Fibonacci number recursive series. Overall, results from the continuous model provide measures of discrete system dynamics including temporal evolution of I and M cell numbers and growth of model structures.

## 4 Discussion

The main finding of our modeling of asymmetric cell division is that a simple set of temporal and spatial rules (see Table 1) can generate patterns of cell populations that maintain their organization while continuing to dynamically self‐renew, which may explain the fidelity of colon tissue renewal. How tissue organization is maintained during tissue renewal, to our knowledge, is a question that has not been extensively pursued. Our previous work (see [Boman et al. 2017]) accounted for the numbers of three different cell types. Our work herein expands those ideas by incorporating spatial organization resulting from rotation and order of cell division. In addition, we account for three‐dimensional organization, while models in (Boman et al. 2017) involved only two‐dimensional tree structures. A modeling challenge is that in histology, microscopic tissue sections show that tissue organization continually stays constant, making tissues appear static, when in fact tissues are fluid systems, incessantly undergoing renewal.

The simple rules addressed in Table 1 are: (1) Timing of cell division, (2) Temporal order of cell division, (3) Spatial direction of cell division, (4) Number of cell divisions, and (5) Cell lifespan. Although the order of cell division may have some variability due to factors such as environmental ones and disease, we hypothesize that, for tissues to renew themselves innumerable times during the lifetime of an organism, the renewal process has to follow a temporal order of cell division. Indeed, the adult male *Caenorhabditis elegans* has exactly 1031 somatic cells, and precise organization of these cells is dynamically conserved across all individuals.

Another result from modeling asymmetric cell division is that model output produces cell numbers and organizational patterns that fit with patterns of organization universally found in nature. Specifically, our model output fits a well‐known mathematical recursive series—the generalized Fibonacci sequences. Indeed, it is well‐known that Fibonacci numbers provide a quantitative description of the phenotype of many living organisms (Knott 2010; Rozin 2020). Even though Fibonacci numbers frequently appear in patterns of growth of plants and animals in nature, the biologic mechanism responsible for the existence of these patterns has not been fully elucidated. The Fibonacci patterns are so common in nature that it seems unlikely that they occur randomly or by chance. Even the geometric proportions of the human body can be described by ratio of Fibonacci numbers (termed the “golden ratio”) (Knott 2010; Rozin 2020). Fibonacci number patterns have also been reported to occur at intracellular and cellular scales including the organization of nucleic acid bases in DNA, (termed the “DNA supra code”; [Perez 2015]), the replication of DNA (Robertson 2001), and clonal growth of human epithelial cells in vitro (Wille 2012). This appearance of Fibonacci numbers at different scales—molecular, cellular, organismic—when viewed from the concept of self‐similarity suggests that a common underlying mechanism for these Fibonacci patterns exists. In this view, our spatial‐temporal asymmetric division mechanism model might lead to an understanding of how Fibonacci numbers arise in patterns of growth of plants and animals and how living organisms are structured at different scales.

Additional key assumptions from our modeling are: (a) daughter cells must inherit instructions for timing, temporal order, and spatial direction of cell division, and (b) the direction of cell division is linked to the maturation period. The link between the maturation period and the spatial direction of cell division (*R*) was necessitated by our observation that, of the numerous model designs we created, the only design that simulated self‐renewing tissue organization was the design that incorporated such a link. The production of patterns of cell populations that maintain their organization while continuing to self‐renew shows that model output can simulate cell dynamics present in biologic tissues. For example, our modeling of self‐renewing tissue organization (Figure 2A–E) shows that model output simulates the biology of human colonic epithelial tissue organization and structure (Figure 2C,F–J). In this line of thinking, the same set of rules may generate organizational patterns that simulate the organization of colonic crypts seen in cross and longitudinal sections of intestinal epithelium (Figure 2C,F). The same set of rules might also provide a mechanism that helps explain the coordination between crypt fission and crypt renewal processes that dynamically maintains organization of the single layer of cells (i.e., columnar epithelium) and establishes colon tissue histology.

Indeed, the growth and maintenance of the model structures—which are generated from model output—occurred due to ongoing cell division within the structure (simulating self‐renewal), not just at the ends or edges of the structure (as in crystal growth). Even growth of leaves of plants occurs by cell division throughout the leaf, including within its interior, which gives rise to its specific size and shape. Thus, our results may explain the biology of tissue renewal because in the maintenance of tissues, cells are constantly dividing in regions of active proliferation throughout the tissue while the organization of cells in tissues stays exactly the same.

What biological evidence supports the possibility of a cellular rotational mechanism as proposed in the model? A major feature of our model design is that cells must rotate in a precise coordinated fashion to generate branches that maintain their organization. In biology, there are many known intracellular rotational mechanisms which could serve as the link to the angle of cell division. For example, motor proteins such as ATP synthase rotate 120° (Imamura et al. 2003; Noji and Yoshida 2001) and centrosomes and the mitotic spindle rotate at specific angles (usually 90°) during the cell cycle (Adams 1996; Gillies and Cabernard 2011; Geldmacher‐Voss et al. 2003; Morin and Bellaiche 2011). Studies of the motion of cells grown in 2D and 3D cultures also show that cells rotate in vitro. When grown on circular 2D substrates (e.g., coated with fibronectin), clusters of cells (from two to several) will coherently rotate together in the absence of any external cues (Brangwynne et al. 2000; Huang et al. 2005; Rørth 2012). However, when grown in standard adherent 2D tissue culture conditions, cells do not appear to rotate, but they will round up during mitosis and become less firmly attached to the culture substratum. When rounded up, mitotic cells would be less constrained and then have the ability to rotate. When grown in 3D cultures, single cells undergo coordinated rotational movement and even cell clusters up to the four cell stage will continue to cohesively rotate (Rørth 2012; Tanner et al. 2012; Wang et al. 2013). Notably, malignant cells do not display this rotational motion in 3D culture. Mathematical models have been created to explain the mechanics of this rotation of cells grown in 2D and 3D culture conditions (Li and Sun 2014; Marmaras et al. 2010; Albert and Schwarz 2016); but they do not explain, as our model does, how the organization of cells in tissues is maintained during tissue renewal.

What biological evidence supports the idea that the direction of division is linked to the maturation period? Finding the answer to this question involves consideration of two biologic processes: (i) how dividing cells in tissues rotate and (ii) how cells coordinate timing and orientation of cell division. The mechanisms underlying these processes are complex and difficult to quantify because epithelial cells exist in tightly inter‐connected epithelial cell sheets in vivo that should constrain rotational motility. Still, as early as 1967 it was observed that the orientation of mitotic figures is different in various epithelia (Doig and Smart 1967) and it has now been well‐substantiated that orientation of epithelial cells during mitosis is tightly controlled in vivo. For example, the orientation of mitosis in the intestine, retina, thyroid, and brain is highly precise and regulated in the process called “elevator movement”, which involves the coordination of many cellular mechanisms (Potten et al. 1988; Jinguji and Ishikawa 1992; Saito et al. 2003; Fujita 2014). This process begins with detachment of mitotic cells from the basal lamina in prophase, which gives them the ability to rotate, and the process ends with reattachment of the daughter cells to the basal lamina in late metaphase. During this process, mitotic cells round up (reminiscent of mitotic cells in 2D cultures) and precisely orient their mitotic spindle, rotate, divide in a specific orientation, and daughter cells re‐insert themselves into particular positions within the epithelium. This precise timing and orientation of cell division in biology might begin to be explained by our modeling that cells inherit instructions for timing, temporal order, and spatial direction of cell division. Indeed, inheritance of these instructions was required for the emergent behavior of cells in order for us to explain how the organization of cells in tissues is precisely maintained.

Another key question is: How does model output, which generates pinwheel structures, fit with similar patterns of cells in nature? It is incorporation of rotation of cell division that generates model pinwheel structures (see Figures 1 and 3). This result simulates the arrangement of rosettes found in normal adult tissues, the classic example being rosettes organized as repeating units in the sub‐ventricular zone (SVZ) of the brain (Mirzadeh et al. 2008). Rosettes in the SVZ reside in the neural stem cell niche which is an area of high neurogenic activity that generates new neurons in adults. Even in embryonic stem cell differentiation, the formation of rosette structures is a hallmark of differentiation along the neural lineage (Elkabetz et al. 2008; Wippold and Perry 2006) and other specialized tissue types (Harding et al. 2014). Thus, our model pinwheel structures that are continuously renewing while in steady state, might fit with the biology of rosettes, which are responsible for tissue renewal in neurogenesis.

Although validating that a link exists between the maturation period (*c* value) and the direction of cell division in tissues in vivo seems quite challenging, there is some evidence that this link exists. For example, the cell cycle time of enterocytes in the human colonic crypt stem cell niche is long (Boman et al. 2001, 2008), and the mitotic spindle of dividing cells aligns perpendicular to the crypt apical surface (Quyn et al. 2010). In contrast, cell cycle time shortens as proliferative cells migrate up the crypt and the mitotic spindle aligns parallel to the apical surface. Such biological findings indicate the direction of cell division is linked to cell cycle time.

What molecular mechanisms might link these spatial and temporal processes involved in cell division (i.e., maturation period and direction of cell division)? The most logical answer is that it involves a mechanism that links intracellular processes such as large scaffold proteins and hub‐type protein complexes that interconnect temporal and spatial processes involved in cell division. For example, the APC and AXIN tumor suppressor proteins are large scaffold proteins that interconnect mitotic spindle orientation and transcriptional regulation, which is crucial to the maintenance of the organization of cell populations in the human colonic crypt (Boman and Fields 2013; Zhang et al. 2010; Huang et al. 2009; Emerick et al. 2017; Emerick et al. 2018). Notably, network hub scaffold‐type proteins are encoded by many cancer genes, which otherwise, if not mutant, normally function by linking different cellular processes such as proliferation and differentiation (Wang et al. 2007; Xu and Fang 2015; Kar et al. 2009).

It is worth noting that there are other processes that can affect cell turnover in the colon. For example, intestinal enteroendocrine cells can regulate crypt cell proliferation through neural signaling by the enteric nervous system, the autonomic nervous system, or the central nervous system (via the brain gut axis) (Farhadi et al. 2007; Farhadi et al. 2005). Also, enteroendocrine cells can act as sensory cells by detecting various nutrients and releasing specific neuropeptides that can either stimulate or inhibit cell proliferation depending on the detected signal (Sternini et al. 2008; Worthington et al. 2018; Modarai et al. 2016; Zhang et al. 2020; Modarai et al. 2018). Moreover, the intestinal environment and mucosal immunity can impact cell division in response to gut microbiota, amino acids, and changes in pH (Di Luccia et al. 2022; Kong et al. 2018). Additionally, the rate of apoptosis and cell extrusion at the mucosal surface can be affected by inflammatory responses leading to changes in homeostasis of colonic epithelium (Blander 2016; Ramachandran et al. 2000; Williams et al. 2015). Nevertheless, the aforementioned pathophysiological responses can only be explained in relation to changes in normal epithelial structure indicating that it is the dynamic organization of cells in colonic epithelium that is fundamental to its basic form and function.

Finally, we did this study not only because tissue organization is an important biological phenomenon in it its own right, but also, because identifying mechanisms that cause tissue disorganization might explain tumorigenesis. Specifically, we believed that to really understand cancer development, we must understand what controls the organization of normal, healthy tissues. Moreover, elucidation of a tissue code could bridge the gap between the pathologists’ and the geneticists’ perspectives on cancer development. From the perspective of pathologists, tissue disorganization is the root of cancer (Foulds 1969; Sonnenschein and Soto 1999; Chun‐Chao et al. 2012; Hinck and Nathke 2014). Indeed, cancer diagnosis universally rests on microscopic evidence of tissue disorganization, invasion, and metastasis. From the geneticists’ perspective, on the other hand, cancer is caused by gene mutations. In fact, based on The Cancer Genome Atlas Project, any given cancer carries large numbers of mutations (100s to 1000s) and other molecular alterations (Stratton et al. 2009; Alexandrov et al. 2013; Vogelstein et al. 2013). Combining these two perspectives, one can argue that tissue disorganization and genetic alterations are both necessary for cancer development, but neither is sufficient to explain cancer. Thus, identification of a tissue code, and understanding how disrupting that code gives rise to tissue pathology, might provide a mechanism that helps explain how gene mutations lead to tissue disorganization and subsequently cancer.

It could also help understand mechanisms that lead to tissue disorganization which contribute to other diseases. Indeed, it is unlikely that tissues can tolerate many errors in their cellular organization without suffering serious impairment of function. One example is disorders of bone structure due to aberrant renewal and remodeling (e.g., osteoporosis). Another example is metaplasia in Barrett's esophagus. In that disorder, esophageal epithelium becomes changed into a different epithelial type (intestinal) that predisposes to cancer (Rustgi and El‐Serag 2014). Indeed, our model predicts that if daughter cells inherit disordered instructions (altered rules), it will lead to tissue disorganization. Therefore, our findings may help us understand how the code for healthy tissue, that is, the *tissue code*, becomes disrupted in a way that leads to tissue disorganization and to pathology.

## 5 Conclusion

Based on modeling of temporal and spatial asymmetries of cell division in the colonic crypt, we put forward the idea that just a few simple rules may explain how living organisms maintain themselves in a highly ordered state despite continuous renewal within their tissues. The “tissue code” could also help understand how the phenotype of an organism emerges through expression of its genes and how tissue pathology (such as cancer) arises from genetic alterations. Therefore, in addition to the genetic code that explains fidelity to genotypic expression, there may well be a code that explains the fidelity of tissue organization.

## Author Contributions

B.M. Boman: designed research, performed research, analyzed data, wrote the paper. T.‐N. Dinh: performed research, contributed computational analytic tools, analyzed data, contributed to writing the paper. K. Decker: performed research, contributed computational analytic tools, analyzed data. B. Emerick: performed research, contributed computational analytic tools, analyzed data. J.Z. Fields: analyzed data, significantly contributed to writing the paper. C. Raymond: analyzed data, contributed to writing the paper. G. Schleiniger: designed research, performed research, contributed computational analytic tools, analyzed data, contributed to writing the paper.

## Conflicts of Interest

The authors declare no conflicts of interest.

## Supporting Data

Supporting data on Fibonacci *p*‐number sequences can be accessed at OEIS.org

## Supporting information




**Supporting file 1**: boc70017‐sup‐0001‐SuppMat.pdf.
