# Supplementary material for: Dynamic Organization of Cells in Colonic Epithelium is Encoded by Five Biological Rules
Source: Biol Cell. 2025 Jul 14;117(7):e70017. doi: 10.1111/boc.70017 (PMC12258116; doi:10.1111/boc.70017)
Supplement: Supplementary file 1 — Supporting file 1: boc70017‐sup‐0001‐SuppMat.pdf. [file BOC-117-e70017-s001.pdf]

Supplemental Materials

Materials and Methods.

Experimental Design

We created two mathematical models (one discrete, the other continuous) based on asymmetric cell division. Our discrete model accounts for each cell in the system with rules for timing and direction of cell division and was programed as an agent-based code. Our continuous model assumes a large number of cells, and accounts for the relative proportion of each cell type.

Agent-based model code for the discrete model

In the code, the sum of cell properties and rules defined the analytical description of the model, which was encoded into NetLogo software used to generate output for the actual simulation. In this code, cells have a position that is defined by their place within an implicit tree data structure that is rooted at the clonogenic cell. In this tree, all nodes are cells and all edges represent connections to either its immediate neighbors or empty space. In model runs, the split-angle is continually tracked and updated for I and M cells. The computer code is available upon request from the corresponding author.

Continuous model

Our continuous model for the dynamics of the system is described by rate equations and rate constant values for division of mature and immature cells in the system population. Only the time evolution of the population of each cell type is tracked — no spatial dependence was considered. First-order kinetics was assumed in order to derive a simple linear model that gives insight into the initial stages of cell population growth.

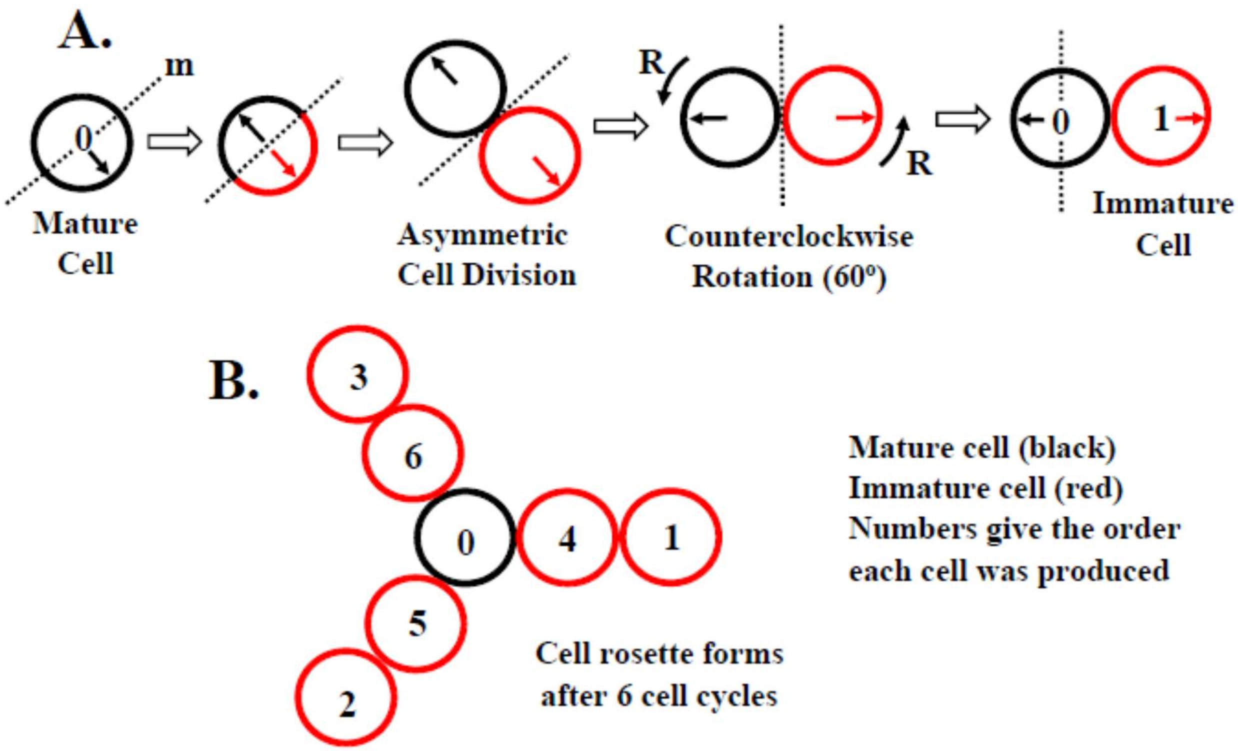

Supplemental Figure 1. Asymmetric division and rotation based on a different set of settings. Panel A shows modeling of cell division based on spatial and temporal asymmetry (Rule 2) according to a simple splitting criterion. Division is modeled as a reflection mechanism determined by a mirror line (m) running through the cell center and perpendicular to the mature cell (black circle) splitting orientation (solid black arrow). After cell division, the mature cell inherits orientation in the opposite direction, while the immature cell (red circle) inherits orientation in the original direction. After cell division, both the mature and immature cells rotate by angle R counterclockwise about their current orientation (Rule 3). After rotation, the mature cell becomes the grid center (i.e., the clonogenic cell) and the immature cell holds its position relative to the center of the grid. Cell division continues with the mature cell and its new mirror line. Cell splitting continues until an immature cell is produced that occupies the space that already contains an immature cell which then becomes displaced one position away and in the appropriate direction. Therefore, the process repeats itself based on the angle of rotation R after cell division and patterns are formed (Panel B).

R (degrees)	R (rads)	ℓ (leaflets)	N (steps)
180	π	1	3
0	0	2	5
60	$\pi/3$	3	7
90	$\pi/2$	4	9
36	$\pi/5$	5	11
120	$2\pi/3$	6	13
45	$\pi/4$	8	17
20	$\pi/9$	9	19
72	$2\pi/5$	10	21
30	$\pi/6$	12	25
12	$\pi/15$	15	31
40	$2\pi/9$	18	37
18	$\pi/10$	20	41
15	$\pi/12$	24	49
24	$2\pi/15$	30	61
10	$\pi/18$	36	73
9	$\pi/20$	40	81
4	$\pi/45$	45	91
6	$\pi/30$	60	121
5	$\pi/36$	72	145
8	$2\pi/45$	90	181
3	$\pi/60$	120	241
2	$\pi/90$	180	361
1	$\pi/180$	360	721

Supplemental Table 1. The orientation of the mature and immature cells. The orientation of the mature cell at step n, denoted by θ_n, follows the sequence below if it is assumed that the initial orientation of the mature cell is 0 radians or oriented toward the positive x-axis.

$$\theta_1 = 0, \quad \theta_2 = \pi, \quad \theta_n = \theta_{n-2} + R + \pi,$$

Using this relation, the spatial position of the i_{th} daughter cell, denoted D_i, can be determined by the following sequence.

$$D_i = \theta_{2i-1} + R, \qquad \text{for } i = 1, 2, \ldots, \frac{N-1}{2}.$$

The i_{th} daughter cell is located on the ray that makes an angle D_i with the x-axis. If the θ in the previous equation is eliminated, the following formula is obtained.

$$D_i = (i-1)\pi + iR, \qquad \text{for } i = 1, 2, \ldots, \ell,$$

where ℓ is the total number of leaflets, $\ell = (N\text{-}1)/2$. Thus, the number of leaflets is related to the given rotational degree, R.

<u>Supplemental Table 2.</u> Generating Function Results for Growing Branch Sizes

Generating Function Results for Growing Branch Size (c = 1)		
**Time**	**Branch AB**	**Total**
0	1	1
1	2	2
2	4	4
3	8	8
4	16	16
5	32	32
6	64	64
7	128	128
8	256	256
9	512	512
10	1024	1024
11	2048	2048
12	4096	4096
13	8192	8192
14	16384	16384

Generating Function Results for Growing Branch Sizes (c = 2)			
**Time**	**Branch AB**	**Branch AC**	**Total**
0	1	1	2
1	2	1	3
2	3	2	5
3	5	3	8
4	8	5	13
5	13	8	21
6	21	13	34
7	34	21	55
8	55	34	89
9	89	55	144
10	144	89	233
11	233	144	377
12	377	233	610
13	610	377	987
14	987	610	1597

Generating Function Results for Growing Branch Sizes (c = 3)				
**Time**	**Branch AB**	**Branch AC**	**Branch AD**	**Total**
0	1	1	1	3
1	2	1	1	4
2	3	2	1	6
3	4	3	2	9
4	6	4	3	13
5	9	6	4	19
6	13	9	6	28
7	19	13	9	41
8	28	19	13	60
9	41	28	19	88
10	60	41	28	129
11	88	60	41	189
12	129	88	60	277
13	189	129	88	406
14	277	189	129	595

Generating Function Results for Growing Branch Sizes (c = 4)					
**Time**	**Branch AB**	**Branch AC**	**Branch AD**	**Branch AE**	**Total**
0	1	1	1	1	4
1	2	1	1	1	5
2	3	2	1	1	7
3	4	3	2	1	10
4	5	4	3	2	14
5	7	5	4	3	19
6	10	7	5	4	26
7	14	10	7	5	36
8	19	14	10	7	50
9	26	19	14	10	69
10	36	26	19	14	95
11	50	36	26	19	131
12	69	50	36	26	181
13	95	69	50	36	250
14	131	95	69	50	345

Generating Function Results for Growing Branch Sizes (c = 5)						
**Time**	**Branch AB**	**Branch AC**	**Branch AD**	**Branch AE**	**Branch AF**	**Total**
0	1	1	1	1	1	5
1	2	1	1	1	1	6
2	3	2	1	1	1	8
3	4	3	2	1	1	11
4	5	4	3	2	1	15
5	6	5	4	3	2	20
6	8	6	5	4	3	26
7	11	8	6	5	4	34
8	15	11	8	6	5	45
9	20	15	11	8	6	60
10	26	20	15	11	8	80
11	34	26	20	15	11	106
12	45	34	26	20	15	140
13	60	45	34	26	20	185
14	80	60	45	34	26	245

Supplemental Figure 2. **A simple continuous model**

A simple linear kinetic model of asymmetric cell division is described by the kinetic equations

$$\mathrm{M} \xrightarrow{k_1} \mathrm{M} + \mathrm{I} \qquad\qquad \mathrm{I} \xrightarrow{k_2} \mathrm{M} + \mathrm{I}$$

$$\mathrm{I} \xrightarrow{k_3} \mathrm{W_1} \qquad\qquad \mathrm{M} \xrightarrow{k_4} \mathrm{W_2}$$

$$\mathrm{W_1} \xrightarrow{k_5} \text{out} \qquad\qquad \mathrm{W_2} \xrightarrow{k_6} \text{out}$$

It follows that the differential equations governing the concentrations of mature, immature and wholly-mature cells are

$$\frac{dM}{dt} = k_2\, I - k_4\, M\,, \qquad \frac{dI}{dt} = k_1\, M - k_3\, I \tag{1}$$

$$\frac{dW_1}{dt} = k_3\, I - k_5\, W_1\,, \qquad \frac{dW_2}{dt} = k_4\, M - k_6\, W_2 \tag{2}$$

Let $\mathbf{c}$ be the concentration vector: $\mathbf{c} = (M, I, W_1, W_2)$. The equivalent system is

$$\frac{d\mathbf{c}}{dt} = A\,\mathbf{c}\,, \quad \text{where} \quad A = \begin{bmatrix} -k_4 & k_2 & 0 & 0 \\ k_1 & -k_3 & 0 & 0 \\ 0 & k_3 & -k_5 & 0 \\ k_4 & 0 & 0 & -k_6 \end{bmatrix}, \tag{3}$$

and the characteristic equation is

$$\left[\lambda^2 + (k_3 + k_4)\lambda + k_3k_4 - k_1k_2\right](\lambda + k_5)(\lambda + k_6) = 0.$$

The eigenvalues are

$$\lambda_{1,2} = \frac{-(k_3 + k_4) \pm \sqrt{(k_3 + k_4)^2 - 4(k_3k_4 - k_1k_2)}}{2}\,, \quad \lambda_3 = -k_5\,, \quad \lambda_4 = -k_6\,.$$

Hence, three eigenvalues are negative ($\lambda_2 < 0\,,\ \lambda_3 < 0$ and $\lambda_4 < 0$) and the other, λ_1 ($\lambda_1 > \lambda_2$), is zero, positive or negative depending on whether $k_3k_4 = k_1k_2\,,\ k_3k_4 < k_1k_2$ or $k_3k_4 > k_1k_2\,,$ respectively. In order to avoid exponential growth, $k_3k_4 \geq k_1k_2$ must hold.

The solution can be easily obtained in terms of the initial values of the cell populations, $M_0\,,\ I_0\,,$ $W_{1,0}$ and $W_{2,0}$. Let

$$K = \sqrt{(k_3 + k_4)^2 - 4(k_3k_4 - k_1k_2)} = \sqrt{(k_3 - k_4)^2 + 4k_1k_2}\,.$$

The general solution of (1),(2) is

$$M(t) = 2k_2\left(c_1\, e^{\lambda_1 t} + c_2\, e^{\lambda_2 t}\right), \quad I(t) = c_1\,(k_4 - k_3 + K)\, e^{\lambda_1 t} + c_2\,(k_4 - k_3 - K)\, e^{\lambda_2 t}, \tag{4}$$

$$W_1(t) = k_3\left(c_1\,\frac{k_4 - k_3 + K}{\lambda_1 + k_5}\, e^{\lambda_1 t} + c_2\,\frac{k_4 - k_3 - K}{\lambda_2 + k_5}\, e^{\lambda_2 t}\right) + c_3\, e^{\lambda_3 t}, \tag{5}$$

$$W_2(t) = 2k_2k_4\left(\frac{c_1}{\lambda_1 + k_6}\, e^{\lambda_1 t} + \frac{c_2}{\lambda_2 + k_6}\, e^{\lambda_2 t}\right) + c_4\, e^{\lambda_4 t}, \tag{6}$$

where the constants c_1 , c_2 , c_3 and c_4 are determined from the initial populations. The expressions (4)–(6) assume that λ_1 and λ_2 are distinct from both λ_3 and λ_4 . If this is not the case, similar expressions are obtained and the behavior discussed below is the same.

It is convenient to non-dimensionalize time by $1/k_1$, the characteristic time for mature cell division, i.e. time will be measured in units of $1/k_1$. This is equivalent to replacing k_j by k_j/k_1 in equation (1),(2), or setting $k_1 = 1$ throughout.

In all cases, the ratio of mature to immature cells in the long run is

$$\frac{M(t)}{I(t)} \approx \frac{2k_2}{K + k_4 - k_3} = \frac{2k_2}{k_4 - k_3 + \sqrt{(k_4 - k_3)^2 + 4k_2}} \,. \tag{7}$$

The constants in (4)–(6) are

$$c_1 = \frac{2k_2 I_0 + (k_3 - k_4 + K)\, M_0}{4k_2 K} \,, \quad c_2 = \frac{(k_4 - k_3 + K)\, M_0 - 2k_2 I_0}{4k_2 K} \,, \tag{8}$$

$$c_3 = W_{1,0} - \frac{k_3}{2K} \left[\frac{(K + k_4 - k_3) I_0 + 2M_0}{\lambda_1 + k_5} + \frac{(K + k_3 - k_4) I_0 - 2M_0}{\lambda_2 + k_5} \right] , \tag{9}$$

$$c_4 = W_{2,0} - \frac{k_4}{2K} \left[\frac{2k_2 I_0 + (k_3 - k_4 + K)\, M_0}{\lambda_1 + k_6} + \frac{(k_4 - k_3 + K)\, M_0 - 2k_2 I_0}{\lambda_2 + k_6} \right] . \tag{10}$$

The limiting relative ratios (for large t) are

$$\frac{M(t)}{I(t)} \approx \frac{2k_2}{k_4 - k_3 + K} \,, \quad \frac{W_2(t)}{M(t)} \approx \frac{2k_4}{2k_6 - k_3 - k_4 + K} \,, \quad \frac{W_1(t)}{I(t)} \approx \frac{2k_3}{2k_5 - k_3 - k_4 + K} \,.$$

A nonzero steady-state for this simple model can only exist if $\lambda_1 = 0$, i.e. only if $k_3 k_4 = k_2$, in which case $K = k_3 + k_4$, $\lambda_2 = -(k_3 + k_4)$ and the relative ratios at steady-state are

$$\frac{M(t)}{I(t)} = \frac{k_2}{k_4} = k_3 \,, \quad \frac{W_2(t)}{M(t)} = \frac{k_4}{k_6} \,, \quad \frac{W_1(t)}{I(t)} = \frac{k_3}{k_5} \,.$$

The unique, asymptotically stable, steady-state is given by

$$M(t) = \frac{k_2 I_0 + k_3 M_0}{k_3 + k_4} \,, \quad I(t) = \frac{k_2 I_0 + k_3 M_0}{k_3 (k_3 + k_4)} \,, \tag{11}$$

$$W_1(t) = \frac{k_2 I_0 + k_3 M_0}{k_5 (k_3 + k_4)} \,, \quad W_2(t) = \frac{k_4 (k_2 I_0 + k_3 M_0)}{k_6 (k_3 + k_4)} \,. \tag{12}$$

Modeling the initial stage of tissue formation, prior to any cell reaching whole maturation, is equivalent to setting $k_3 = k_4 = 0$ in (1),(2), and $W_{1,0} = W_{2,0} = 0$, in which case $\lambda_{1,2} = \pm\sqrt{k_2}$, $K = 2\sqrt{k_2}$, and from (8)–(10)

$$c_1 = \frac{1}{4\sqrt{k_2}} \left(\frac{M_0}{\sqrt{k_2}} + I_0 \right) , \quad c_2 = \frac{1}{4\sqrt{k_2}} \left(\frac{M_0}{\sqrt{k_2}} - I_0 \right) , \quad c_3 = c_4 = 0 \,.$$

Hence, in the initial stages of tissue formation, from (4) we obtain

$$M(t) = \sqrt{k_2}\,\frac{e^{\sqrt{k_2}t}}{2}\left[\frac{M_0}{\sqrt{k_2}} + I_0 + \left(\frac{M_0}{\sqrt{k_2}} - I_0\right)e^{-2\sqrt{k_2}t}\right] \tag{13}$$

$$I(t) = \frac{e^{\sqrt{k_2}t}}{2}\left[\frac{M_0}{\sqrt{k_2}} + I_0 - \left(\frac{M_0}{\sqrt{k_2}} - I_0\right)e^{-2\sqrt{k_2}t}\right] \tag{14}$$

so that, even for a relatively short time (in units of characteristic time of mature cell division),

$$\frac{M(t)}{I(t)} \approx \sqrt{k_2}\,.$$

Therefore, consistency between the continuous model and the discrete model requires that this relation holds for the latest times of the initial stage of tissue formation. This is consistent with the idea proposed in the Introduction of a set of biologic rules that encodes information in cells for histologic structure similar to the information contained in the meristem of a plant that determines its specific structure and organization. The initial stages of tissue development are determined by the rates of mature and immature cell division, k_1 and k_2, where $k_1 = 1$ follows from choosing the time scale to be $1/k_1$, and the value of k_2 follows from the rules described in the discrete model.

Supplemental Figure 3: Relationship between discrete model output expressed as a quotient of growing branch sizes for $c = 2$ to $c = 6$ and the k_2 rate constant values from the continuous model.

c = 2

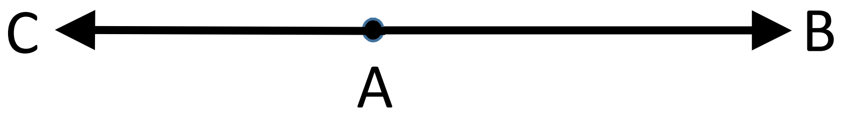

System Relationships	Correlation with k_2 Rate Constant	Value
$\frac{AC}{AB + AC}$	k_2	0.381966
$\frac{AB + AC}{AC}$	$\frac{1}{k_2} = 2+\sqrt[2]{k_2}$	2.618034
$\frac{AC}{AB} = \frac{AB}{CB} = \frac{AB}{AB+AC}$	$\sqrt[2]{k_2} = 1\text{-}k_2$	0.618034
$\frac{AB}{AC} = \frac{CB}{AB} = \frac{AB+AC}{AB}$	$\frac{1}{\sqrt[2]{k_2}} = 1+\sqrt[2]{k_2}$	1.618034

Polynomial Expressions & Root Values*		
k_2	$x^2 - 3x + 1 = 0$	X1 = 2.618034<br>X2 = 0.381967
$\frac{1}{k_2} = 2+\sqrt[2]{k_2}$	$x^2 - 3x + 1 = 0$	X1 = 2.618034<br>X2 = 0.381967
$\sqrt[2]{k_2} = 1\text{-}k_2$	$x^2 + x - 1 = 0$	X1 = 0.618034<br>X2 = -1.618034
$\frac{1}{\sqrt[2]{k_2}} = 1+\sqrt[2]{k_2}$	$x^2 - x - 1 = 0$	X1 = 1.618034<br>X2 = -0.618034

*See Table S3 for exact root values

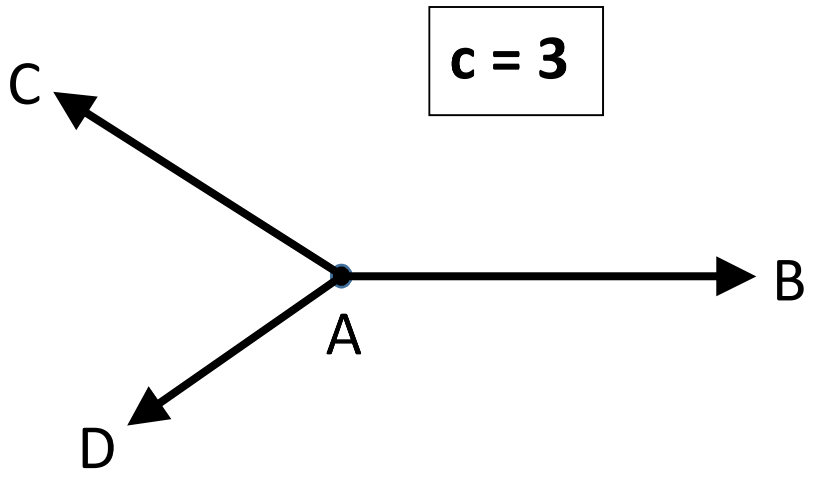

System Relationships	Correlation with k_2 Rate Constant	Value
$\frac{AD}{AB + AC + AD}$	k_2	0.216757
$\frac{AD}{AB} = \frac{AC}{AB+AD} = \frac{AB}{AB+AC+AD}$	$\sqrt[2]{k_2}$	0.465571
$\frac{AB}{AD} = \frac{AB+AC+AD}{AB}$	$\frac{1}{\sqrt[2]{k_2}}$	2.147900
$\frac{AC}{AB} = \frac{AD}{AC} = \frac{AB}{AB+AD}$	$\sqrt[4]{k_2}$	0.682328
$\frac{AB}{AC} = \frac{AC}{AD} = \frac{AB+AD}{AB}$	$\frac{1}{\sqrt[4]{k_2}} = 1 + \sqrt[2]{k_2}$	1.465571
$\frac{AC}{AB+AC+AD} = \frac{AD}{AB+AD}$	$1 - \sqrt[4]{k_2}$	0.317672
$\frac{AB+AC+AD}{AC} = \frac{AB+AD}{AD}$	$\frac{1}{1 - \sqrt[4]{k_2}} = 1 + \frac{1}{\sqrt[2]{k_2}}$	3.1479

Polynomial Expressions & Root Values*		
k_2	$x^3 - 2x^2 + 5x - 1 = 0$	$X_1 = 0.21676$<br>$X_2 = 0.89162+1.95409*i$<br>$X_3 = 0.89162-1.95409*i$
$\sqrt[2]{k_2}$	$x^3 + 2x^2 + x - 1 = 0$	$X_1 = 0.46557$<br>$X_2 = -1.23279+0.79255*i$<br>$X_3 = -1.23279-0.79255*i$
$\frac{1}{\sqrt[2]{k_2}}$	$x^3 - x^2 - 2x - 1 = 0$	$X_1 = 2.1479$<br>$X_2 = -0.57395+0.36899*i$<br>$X_3 = -0.57395-0.36899*i$
$\sqrt[4]{k_2}$	$x^3 + x - 1 = 0$	$X_1 = 0.68233$<br>$X_2 = -0.34116+1.16154*i$<br>$X_3 = -0.34116-1.16154*i$
$\frac{1}{\sqrt[4]{k_2}} = 1 + \sqrt[2]{k_2}$	$x^3 - x^2 - 1 = 0$	$X_1 = 1.46557$<br>$X_2 = -0.23279+0.79255*i$<br>$X_3 = -0.23279-0.79255*i$
$1 - \sqrt[4]{k_2}$	$x^3 - 3x^2 + 4x - 1 = 0$	$X_1 = 0.31767$<br>$X_2 = 1.34116+1.16154*i$<br>$X_3 = 1.34116-1.16154*i$
$\frac{1}{1 - \sqrt[4]{k_2}} = 1 + \frac{1}{\sqrt[2]{k_2}}$	$x^3 - 4x^2 + 3x - 1 = 0$	$X_1 = 3.1479$<br>$X_2 = 0.42605+0.36899*i$<br>$X_3 = 0.42605-0.36899*i$

*See Table S3 for exact root values

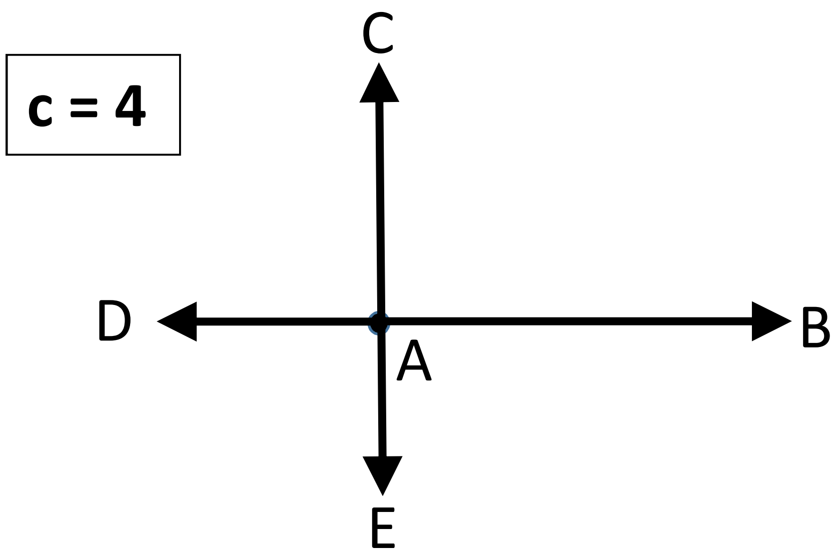

System Relationships	Correlation with k_2 Rate Constant	Value
$\frac{AE}{AB + AC + AD + AE}$	k_2	0.144611
$\frac{AE}{AB} = \frac{AB}{AB+AC+AD+AE} = \frac{AC}{AB+AD+AE} = \frac{AD}{AB+AE}$	$\sqrt[2]{k_2}$	0.380277
$\frac{AB}{AE} = \frac{AB+AC+AD+AE}{AB} = \frac{AB+AD+AE}{AC} = \frac{AB+AE}{AD}$	$\frac{1}{\sqrt[2]{k_2}}$	2.629662
$\frac{AC}{AB} = \frac{AD}{AC} = \frac{AE}{AD} = \frac{AB}{AB+AE}$	$\sqrt[6]{k_2}$	0.724491
$\frac{AB}{AC} = \frac{AC}{AD} = \frac{AD}{AE} = \frac{AB+AE}{AB}$	$\frac{1}{\sqrt[6]{k_2}} = 1 + \sqrt[2]{k_2}$	1.380277

$\frac{AC}{AB+AC+AD+AE} = \frac{AD}{AB+AD+AE} = \frac{AE}{AB+AE}$	$1 - \sqrt[6]{k_2}$	0.275508
$\frac{AB+AC+AD+AE}{AC} = \frac{AB+AD+AE}{AD} = \frac{AB+AE}{AE}$	$\frac{1}{1-\sqrt[6]{k_2}} = 1 + \frac{1}{\sqrt[2]{k_2}}$	3.629659
$\frac{AD}{AB} = \frac{AE}{AC}$	$\sqrt[3]{k_2}$	0.524888
$\frac{AB}{AD} = \frac{AC}{AE}$	$\frac{1}{\sqrt[3]{k_2}}$	1.905168

Polynomial Expressions & Root Values* for c = 4		
k_2	$x^4 - 3x^3 + x^2 - 7x + 1 = 0$	X_1 = 0.144611<br>X_2 = 3.30939<br>X_3 = -0.227+1.42759*i<br>X_4 = -0.227-1.42759*i
$\sqrt[2]{k_2}$	$x^4 + 3x^3 + 3x^2 + x - 1 = 0$	X_1 = 0.380277<br>X_2 = -1.81917<br>X_3 = -0.78055+0.91447*i<br>X_4 = -0.78055-0.91447*i
$\frac{1}{\sqrt[2]{k_2}}$	$x^4 - x^3 - 3x^2 - 3x - 1 = 0$	X_1 = -0.5497<br>X_2 = 2.62966<br>X_3 = -0.53998+0.63262*i<br>X_4 = -0.53998-0.63262*i

$\sqrt[6]{k_2}$	$x^4 + x - 1 = 0$	**X_1 = 0.724491**<br>**X_2 = -1.22074**<br>**X_3 = 0.24813+1.03398*i**<br>**X_4 = 0.24813-1.03398*i**
$\frac{1}{\sqrt[6]{k_2}} = 1 + \sqrt[2]{k_2}$	$x^4 - x^3 - 1 = 0$	**X_1 = -0.81917**<br>**X_2 = 1.38028**<br>**X_3 = 0.21945+0.91447*i**<br>**X_4 = 0.21945-0.91447*i**
$1 - \sqrt[6]{k_2}$	$x^4 - 4x^3 + 6x^2 - 5x + 1 = 0$	**X_1 = 0.275508**<br>**X_2 = 2.22074**<br>**X_3 = 0.75187+1.03398*i**<br>**X_4 = 0.75187-1.03398*i**
$\frac{1}{1 - \sqrt[6]{k_2}} = 1 + \frac{1}{\sqrt[2]{k_2}}$	$x^4 - 5x^3 + 6x^2 - 4x + 1 = 0$	**X_1 = 0.4503**<br>**X_2 = 3.62966**<br>**X_3 = 0.46002+0.63262*i**<br>**X_4 = 0.46002-0.63262*i**
$\sqrt[3]{k_2}$	$x^4 - 2x^2 - x + 1 = 0$	**X_1 = 0.524888**<br>**X_2 = 1.49022**<br>**X_3 = -1.00755+0.51312*i**<br>**X_4 = -1.00755-0.51312*i**

$\frac{1}{\sqrt[3]{k_2}}$	$x^4 - x^3 - 2x^2 + 1 = 0$	**X_1 = 0.67104**<br>**X_2 = 1.90517**<br>**X_3 = -0.7881+0.40136*i**<br>**X_4 = -0.7881-0.40136*i**

*Exact expressions for the roots of the fourth degree polynomials are available, but their long and complicated expressions are not of special interest for this work.

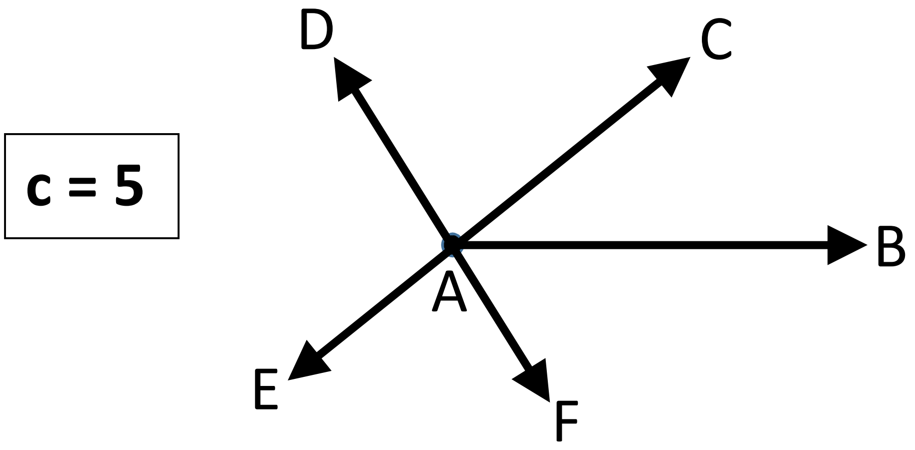

System Relationships	Correlation with k_2 Rate Constant	Value
$\frac{AF}{AB + AC + AD + AE + AF}$	k_2	0.1054418
$\frac{AB}{AB+AC+AD+AE+AF} = \frac{AC}{AB+AD+AE+AF} = \frac{AD}{AB+AE+AF} = \frac{AE}{AB+AF} = \frac{AF}{AB}$	$\sqrt[2]{k_2}$	0.324718
$\frac{AB+AC+AD+AE+AF}{AB} = \frac{AB+AD+AE+AF}{AC} = \frac{AB+AE+AF}{AD} = \frac{AB+AF}{AE} = \frac{AB}{AF}$	$\frac{1}{\sqrt[2]{k_2}}$	3.079595
$\frac{AC}{AB} = \frac{AD}{AC} = \frac{AE}{AD} = \frac{AF}{AE} = \frac{AB}{AB+AF}$	$\sqrt[8]{k_2}$	0.754878
$\frac{AB}{AC} = \frac{AC}{AD} = \frac{AD}{AE} = \frac{AE}{AF} = \frac{AB+AF}{AB}$	$\frac{1}{\sqrt[8]{k_2}} = 1 + \sqrt[2]{k_2}$	1.324718

$\frac{AC}{AB+AC+AD+AE+AF} = \frac{AD}{AB+AD+AE+AF} = \frac{AE}{AB+AE+AF} = \frac{AF}{AB+AF}$	$1 - \sqrt[8]{k_2}$	0.245122
$\frac{AB+AC+AD+AE+AF}{AC} = \frac{AB+AD+AE+AF}{AD} = \frac{AB+AE+AF}{AE} = \frac{AB+AF}{AF}$	$\frac{1}{1-\sqrt[8]{k_2}} = 1+\frac{1}{\sqrt[2]{k_2}}$	4.07960
$\frac{AD}{AB} = \frac{AE}{AC} = \frac{AF}{AD}$	$\sqrt[4]{k_2} = \frac{1}{1+\sqrt[8]{k_2}}$	0.569840
$\frac{AB}{AD} = \frac{AC}{AE} = \frac{AD}{AF}$	$\frac{1}{\sqrt[4]{k_2}} = 1+\sqrt[8]{k_2}$	1.754878

Polynomial Expressions & Root Values*		
k_2	$x^5 - 4x^4 + 6x^3 + 4x^2 + 9x - 1 = 0$	**X_1 = 0.10544**<br>**X_2 = -0.5+0.86603*i**<br>**X_3 = -0.5-0.86603*i**<br>**X_4 = 2.44728+1.86942*i**<br>**X_5 = 2.44728-1.86942*i**
$\sqrt[2]{k_2}$	$x^5 + 4x^4 + 6x^3 + 4x^2 + x - 1 = 0$	**X_1 = 0.32472**<br>**X_2 = -0.5+0.86603*i**<br>**X_3 = -0.5-0.86603*i**<br>**X_4 = -1.66236+0.56228*i**<br>**X_5 = -1.66236-0.56228*i**
$\frac{1}{\sqrt[2]{k_2}}$	$x^5 - x^4 - 4x^3 - 6x^2 - 4x - 1 = 0$	**X_1 = 3.0796**<br>**X_2 = -0.5398+0.18258*i**

		X_3 = -0.5398-0.18258*i<br>X_4 = -0.5+0.86603*i<br>X_5 = -0.5-0.86603*i
$\sqrt[8]{k_2}$	$x^5 + x - 1 = 0$	X_1 = 0.75488<br>X_2 = 0.5+0.86603*i<br>X_3 = 0.5-0.86603*i<br>X_4 = -0.87744+0.74486*i<br>X_5 = -0.87744-0.74486*i
$\frac{1}{\sqrt[8]{k_2}} = 1 + \sqrt[2]{k_2}$	$x^5 - x^4 - 1 = 0$	X_1 = 1.32472<br>X_2 = 0.5+0.86603*i<br>X_3 = 0.5-0.86603*i<br>X_4 = -0.66236+0.56228*i<br>X_5 = -0.66236-0.56228*i
$1 - \sqrt[8]{k_2}$	$x^5 - 5x^4 + 10x^3 - 10x^2 + 6x - 1 = 0$	X_1 = 0.24512<br>X_2 = 0.5+0.86603*i<br>X_3 = 0.5-0.86603*i<br>X_4 = 1.87744+0.74486*i<br>X_5 = 1.87744-0.74486*i
$\frac{1}{1 - \sqrt[8]{k_2}} = 1 + \frac{1}{\sqrt[2]{k_2}}$	$x^5 - 6x^4 + 10x^3 - 10x^2 + 5x - 1 = 0$	X_1 = 4.0796<br>X_2 = 0.4602+0.18258*i<br>X_3 = 0.4602-0.18258*i

		X_4 = 0.5+0.86603*i<br>X_5 = 0.5-0.86603*i
$\sqrt[4]{k_2} = \frac{1}{1+\sqrt[8]{k_2}}$	$x^5 + 2x^3 + x - 1 = 0$	X_1 = 0.56984<br>X_2 = -0.5+0.86603*i<br>X_3 = -0.5-0.86603*i<br>X_4 = 0.21508+1.30714*i<br>X_5 = 0.21508-1.30714*i
$\frac{1}{\sqrt[4]{k_2}} = 1+\sqrt[8]{k_2}$	$x^5 - x^4 - 2x^2 - 1 = 0$	X_1 = 1.75488<br>X_2 = 0.12256+0.74486*i<br>X_3 = 0.12256-0.74486*i<br>X_4 = -0.5+0.86603*i<br>X_5 = -0.5-0.86603*i

*See Table S3 for exact root values

c = 6

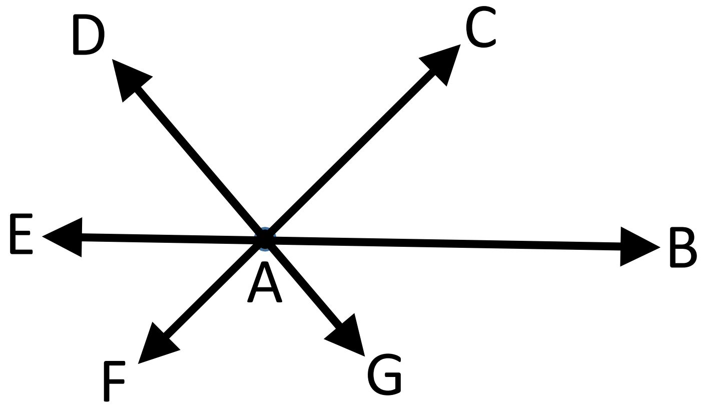

System Relationships	Correlation with k_2 Rate Constant	Value
$\frac{AG}{AB + AC + AD + AE + AF + AG}$	k_2	0.081338
$\frac{AB}{AB+AC+AD+AE+AF+AG} = \frac{AC}{AB+AD+AE+AF+AG} = \frac{AD}{AB+AE+AF+AG} = \frac{AE}{AB+AF+AG} = \frac{AF}{AB+AG} = \frac{AG}{AB}$	$\sqrt[2]{k_2}$	0.285199
$\frac{AB+AC+AD+AE+AF+AG}{AB} = \frac{AB+AD+AE+AF+AG}{AC} = \frac{AB+AE+AF+AG}{AD} = \frac{AB+AF+AG}{AE} = \frac{AB+AG}{AF} = \frac{AB}{AG}$	$\frac{1}{\sqrt[2]{k_2}}$	3.50632
$\frac{AC}{AB} = \frac{AD}{AC} = \frac{AE}{AD} = \frac{AF}{AE} = \frac{AG}{AF}$	$\sqrt[10]{k_2}$	0.77809
$\frac{AB}{AC} = \frac{AC}{AD} = \frac{AD}{AE} = \frac{AE}{AF} = \frac{AF}{AG}$	$\frac{1}{\sqrt[10]{k_2}} = 1 + \sqrt[2]{k_2}$	1.285199
$\frac{AC}{AB+AC+AD+AE+AF+AG} = \frac{AD}{AB+AD+AE+AF+AG} = \frac{AE}{AB+AE+AF+AG} = \frac{AF}{AB+AF+AG} = \frac{AG}{AB+AG}$	$1 - \sqrt[10]{k_2}$	0.22191

$\frac{AB+AC+AD+AE+AF+AG}{AC} = \frac{AB+AD+AE+AF+AG}{AD} = \frac{AB+AE+AF+AG}{AE} = \frac{AB+AF+AG}{AF} = \frac{AB+AG}{AG}$	$\frac{1}{1-\sqrt[10]{k_2}} = 1 + \frac{1}{\sqrt[2]{k_2}}$	4.50633
$\frac{AD}{AB} = \frac{AE}{AC} = \frac{AF}{AD} = \frac{AG}{AE}$	$\sqrt[5]{k_2}$	0.605423
$\frac{AB}{AD} = \frac{AC}{AE} = \frac{AD}{AF} = \frac{AE}{AG}$	$\frac{1}{\sqrt[5]{k_2}}$	1.651737

Polynomial Expressions & Root Values*		
k_2	$x^6 - 5x^5 + 10x^4 - 12x^3 - 15x^2 - 11x + 1 = 0$	**X_1 = 0.08134**<br>**X_2 = 3.53918**<br>**X_3 = -0.50799+0.51585*i**<br>**X_4 = -0.50799-0.51585*i**<br>**X_5 = 1.19773+2.27876*i**<br>**X_6 = 1.19773-2.27876*i**
$\sqrt[2]{k_2}$	$x^6 + 5x^5 + 10x^4 + 10x^3 + 5x^2 + x - 1 = 0$	**X_1 = 0.2852**<br>**X_2 = -1.88127**<br>**X_3 = -0.32863+0.78485*i**<br>**X_4 = -0.32863-0.78485*i**<br>**X_5 = -1.37333+0.82964*i**<br>**X_6 = -1.37333-0.82964*i**
$\frac{1}{\sqrt[2]{k_2}}$	$x^6 - x^5 - 5x^4 - 10x^3 - 10x^2 - 5x - 1 = 0$	**X_1 = -0.53156**<br>**X_2 = 3.50632**

		X_3 = -0.53347+0.32227*i<br>X_4 = -0.53347-0.32227*i<br>X_5 = -0.45392+1.08406*i<br>X_6 = -0.45392-1.08406*i
$\sqrt[10]{k_2}$	$x^6 + x - 1 = 0$	X_1 = 0.77809<br>X_2 = -1.13472<br>X_3 = 0.62937+0.73576*i<br>X_4 = 0.62937-0.73576*i<br>X_5 = -0.45106+1.00236*i<br>X_6 = -0.45106-1.00236*i
$\frac{1}{\sqrt[10]{k_2}} = 1 + \sqrt[2]{k_2}$	$x^6 - x^5 - 1 = 0$	X_1 = -0.88127<br>X_2 = 1.2852<br>X_3 = 0.67137+0.78485*i<br>X_4 = 0.67137-0.78485*i<br>X_5 = -0.37333+0.82964*i<br>X_6 = -0.37333-0.82964*i
$1 - \sqrt[10]{k_2}$	$x^6 - 6x^5 + 15x^4 - 20x^3 + 15x^2 - 7x + 1 = 0$	X_1 = 0.22191<br>X_2 = 2.13472<br>X_3 = 0.37063+0.73576*i<br>X_4 = 0.37063-0.73576*i<br>X_5 = 1.45106+1.00236*i

		X_6 = 1.45106-1.00236*i
$\frac{1}{1-\sqrt[10]{k_2}} = 1+\frac{1}{\sqrt[2]{k_2}}$	$x^6 - 7x^5 + 15x^4 - 20x^3 + 15x^2 - 6x + 1 = 0$	X_1 = 0.46844<br>X_2 = 4.50632<br>X_3 = 0.46653+0.32227*i<br>X_4 = 0.46653-0.32227*i<br>X_5 = 0.54608+1.08406*i<br>X_6 = 0.54608-1.08406*i
$\sqrt[5]{k_2}$	$x^6 - 2x^3 - x + 1 = 0$	X_1 = 0.60542<br>X_2 = 1.2876<br>X_3 = -0.14523+0.92613*i<br>X_4 = -0.14523-0.92613*i<br>X_5 = -0.80128+0.90424*i<br>X_6 = -0.80128-0.90424*i
$\frac{1}{\sqrt[5]{k_2}}$	$x^6 - x^5 - 2x^3 + 1 = 0$	X_1 = 0.77664<br>X_2 = 1.65174<br>X_3 = -0.54893+0.61947*i<br>X_4 = -0.54893-0.61947*i<br>X_5 = -0.16526+1.05385*i<br>X_6 = -0.16526-1.05385*i

*There are no exact root values for these polynomials

Supplemental Table 3: **Exact roots**

$$p_1(x) = x^2 - 3x + 1$$

$$x_1 = \frac{1}{2}(3+\sqrt{5}) \approx 2.618033988\,, \qquad x_2 = \frac{1}{2}(3-\sqrt{5}) \approx 0.381966012$$

$$p_2(x) = x^2 + x - 1$$

$$x_1 = \frac{1}{2}(\sqrt{5}-1) \approx 0..6180339880\,, \quad x_2 = -\frac{1}{2}(1+\sqrt{5}) \approx -1.618033988$$

$$p_3(x) = x^2 - x - 1$$

$$x_1 = \frac{1}{2}(\sqrt{5}+1) \approx 1.618033988\,, \quad x_2 = \frac{1}{2}(1-\sqrt{5}) \approx -0.6180339880$$

$$p_4(x) = x^3 - 2x^2 + 5\,x - 1$$

$$\begin{aligned}
x_1 =& -\frac{1}{6}(188+36\sqrt{93})^{1/3} + \frac{22}{3(188+36\sqrt{93})^{1/3}} + \frac{2}{3} \approx 0.2167565713\,,\\
x_2 =& \frac{1}{12}(188+36\sqrt{93})^{1/3} - \frac{11}{3(188+36\sqrt{93})^{1/3}} + \frac{2}{3}\\
&+ \frac{i}{2}\sqrt{3}\left(-\frac{1}{6}(188+36\sqrt{93})^{1/3} - \frac{22}{3(188+36\sqrt{93})^{1/3}}\right) \approx 0.8916217140 - 1.954093394\,i\,,\\
x_3 =& \frac{1}{12}(188+36\sqrt{93})^{1/3} - \frac{11}{3(188+36\sqrt{93})^{1/3}} + \frac{2}{3}\\
&- \frac{i}{2}\sqrt{3}\left(-\frac{1}{6}(188+36\sqrt{93})^{1/3} - \frac{22}{3(188+36\sqrt{93})^{1/3}}\right) \approx 0.8916217140 + 1.954093394\,i
\end{aligned}$$

$$p_5(x) = x^3 + 2\,x^2 + x - 1$$

$$\begin{aligned}
x_1 =& \frac{1}{6}(116+12\sqrt{93})^{1/3} + \frac{2}{3}\left(\frac{1}{(116+12\sqrt{93})^{1/3}} - 1\right) \approx 0.4655712323\\
x_{2,3} =& -\frac{1}{12}(116+12\sqrt{93})^{1/3} - \frac{1}{3}\left(\frac{1}{(116+12\sqrt{93})^{1/3}} + 2\right)\\
&\pm i\,\frac{\sqrt{3}}{2}\left(\frac{1}{6}(116+12\sqrt{93})^{1/3} - \frac{2}{3(116+12\sqrt{93})^{1/3}}\right) \approx -1.232785616 \pm 0.7925519930\,i
\end{aligned}$$

$$\boldsymbol{p_6(x) = x^3 - x^2 - 2x - 1}$$

$$x_1 = \frac{1}{6}(188 + 12\sqrt{93})^{1/3} + \frac{1}{3}\left(\frac{14}{(188 + 12\sqrt{93})^{1/3}} + 1\right) \approx 2.147899035\,,$$

$$x_{2,3} = -\frac{1}{3}\quad \frac{(188 + 12\sqrt{93})^{1/3}}{4} + \frac{7}{(188 + 12\sqrt{93})^{1/3}} - 1\Bigg)$$

$$\pm\, i\,\frac{\sqrt{3}}{6}\quad \frac{(188 + 12\sqrt{93})^{1/3}}{2} - \frac{14}{(188 + 12\sqrt{93})^{1/3}}\Bigg) \approx 0.5739495177 \pm 0.3689894078\,i$$

$$\boldsymbol{p_7(x) = x^3 + x - 1}$$

$$x_1 = \frac{1}{6}(108 + 12\sqrt{93})^{1/3} - \frac{2}{(108 + 12\sqrt{93})^{1/3}} \approx 0.6823278040\,,$$

$$x_{2,3} = -\frac{1}{12}(108 + 12\sqrt{93})^{1/3} + \frac{1}{(108 + 12\sqrt{93})^{1/3}} \pm i\,\frac{\sqrt{3}}{2}\left(\frac{(108 + 12\sqrt{93})^{1/3}}{6} + \frac{2}{(108 + 12\sqrt{93})^{1/3}}\right)$$

$$\approx -0.3411639019 \pm 1.1615414\,i$$

$$\boldsymbol{p_8(x) = x^3 - x^2 - 1}$$

$$x_1 = \frac{1}{6}(116 + 12\sqrt{93})^{1/3} + \frac{1}{3}\left(\frac{2}{(116 + 12\sqrt{93})^{1/3}} + 1\right) \approx 1.465571232\,,$$

$$x_{2,3} = -\frac{1}{12}(116 + 12\sqrt{93})^{1/3} - \frac{1}{3}\left(\frac{1}{(116 + 12\sqrt{93})^{1/3}} - 1\right)$$

$$\pm\, i\,\frac{\sqrt{3}}{6}\quad \frac{(116 + 12\sqrt{93})^{1/3}}{2} - \frac{2}{(116 + 12\sqrt{93})^{1/3}}\Bigg) \approx -0.2327856159 \pm 0.792551993\,i$$

$$\boldsymbol{p_9(x) = x^3 - 3x^2 + 4\,x - 1}$$

$$x_1 = -\frac{1}{6}(108 + 12\sqrt{93})^{1/3} + \frac{2}{(108 + 12\sqrt{93})^{1/3}} + 1 \approx 0.3176721960\,,$$

$$x_{2,3} = \frac{1}{12}(108 + 12\sqrt{93})^{1/3} - \frac{1}{(108 + 12\sqrt{93})^{1/3}} + 1 \pm i\,\frac{\sqrt{3}}{2}\left(\frac{(108 + 12\sqrt{93})^{1/3}}{6} + \frac{2}{(108 + 12\sqrt{93})^{1/3}}\right)$$

$$\approx 1.341163902 \pm 1.161541400\,i$$

$$p_{10}(x) = x^3 - 4x^2 + 3\,x - 1$$

$$x_1 = \frac{1}{6}(188+12\sqrt{93})^{1/3} + \frac{1}{3}\left(\frac{14}{(188+12\sqrt{93})^{1/3}} + 4\right) \approx 3.147899035\,,$$

$$x_{2,3} = -\frac{1}{12}(188+12\sqrt{93})^{1/3} - \frac{1}{3}\left(\frac{7}{(188+12\sqrt{93})^{1/3}} - 4\right)$$

$$\pm\, i\,\frac{\sqrt{3}}{6}\quad \left. \frac{(188+12\sqrt{93})^{1/3}}{2} - \frac{14}{(188+12\sqrt{93})^{1/3}}\right) \approx 0.4260504820 \pm 0.3689894078\,i$$

$$p_{11}(x) = x^5 - 4x^4 + 6\,x^3 + 4\,x^2 + 9\,x - 1$$

$$x_1 = -\frac{1}{3}\quad \left. \frac{(692+84\sqrt{69})^{1/3}}{2} - \frac{10}{(692+84\sqrt{69})^{1/3}} - 5\right) \approx 0.105441752\,,$$

$$x_{2,3} = \frac{1}{2}(-1 \pm i\,\sqrt{3}) \approx -0.5 \pm 0.8660254040\,i\,,$$

$$x_{4,5} = \frac{1}{3}\left(\frac{(692+84\sqrt{69})^{1/3}}{4} - \frac{5}{(692+84\sqrt{69})^{1/3}} + 5\right) \pm i\,\frac{\sqrt{3}}{6}\quad \left. \frac{(692+84\sqrt{69})^{1/3}}{2} + \frac{10}{(692+84\sqrt{69})^{1/3}}\right)$$

$$\approx 2.447279124 \pm 1.869420791\,i$$

$$p_{12}(x) = x^5 + 4\,x^4 + 6\,x^3 + 4\,x^2 + x - 1$$

$$x_1 = \frac{1}{6}(108+12\sqrt{69})^{1/3} + \frac{2}{(108+12\sqrt{69})^{1/3}} - 1 \approx 0.324717958\,,$$

$$x_{2,3} = \frac{1}{2}(-1 \pm i\,\sqrt{3}) \approx -0.5 \pm 0.8660254040\,i\,,$$

$$x_{4,5} = -\frac{1}{12}(108+12\sqrt{69})^{1/3} - \frac{1}{(108+12\sqrt{69})^{1/3}} - 1 \pm i\,\frac{\sqrt{3}}{2}\quad \left. \frac{(108+12\sqrt{69})^{1/3}}{6} - \frac{2}{(108+12\sqrt{69})^{1/3}}\right)$$

$$\approx -1.662358979 \pm 0.5622795125\,i$$

$$p_{13}(x) = x^5 - x^4 - 4x^3 - 6x^2 - 4x - 1$$

$$\begin{aligned}
x_1 =& \frac{1}{6}(388 + 12\sqrt{69})^{1/3} + \frac{26}{3(388 + 12\sqrt{69})^{1/3}} + \frac{2}{3} \approx 3.079595625 \\
x_{2,3} =& \frac{1}{2}(-1 \pm i\,\sqrt{3}) \approx -0.5 \pm 0.8660254040\,i \\
x_{4,5} =& -\frac{1}{12}(388 + 12\sqrt{69})^{1/3} - \frac{13}{3(388 + 12\sqrt{69})^{1/3}} + \frac{2}{3} \\
& \pm i\,\frac{\sqrt{3}}{2} \quad \frac{(388 + 12\sqrt{69})^{1/3}}{6} - \frac{26}{3(388 + 12\sqrt{69})^{1/3}} \biggr) \approx -0.5397978113 \pm 0.1825822549\,i
\end{aligned}$$

$$p_{14}(x) = x^5 + x - 1$$

$$\begin{aligned}
x_1 =& \frac{1}{6}(100 + 12\sqrt{69})^{1/3} + \frac{2}{3(100 + 12\sqrt{69})^{1/3}} - \frac{1}{3} \approx 0.7548776667\,, \\
x_{2,3} =& \frac{1}{2}(1 \pm i\,\sqrt{3}) \approx 0.5 \pm 0.8660254040\,i\,, \\
x_{4,5} =& -\frac{1}{12}(100 + 12\sqrt{69})^{1/3} - \frac{1}{3(100 + 12\sqrt{69})^{1/3}} - \frac{1}{3} \\
& \pm i\,\frac{\sqrt{3}}{6} \quad \frac{(100 + 12\sqrt{69})^{1/3}}{2} - \frac{2}{(100 + 12\sqrt{69})^{1/3}} \biggr) \approx -0.8774388331 \pm 0.7448617670\,i
\end{aligned}$$

$$p_{15}(x) = x^5 - x^4 - 1$$

$$\begin{aligned}
x_1 =& \frac{1}{6}(108 + 12\sqrt{69})^{1/3} + \frac{2}{(108 + 12\sqrt{69})^{1/3}} \approx 1.324717958\,, \\
x_{2,3} =& \frac{1}{2}(1 \pm i\,\sqrt{3}) \approx 0.5 \pm 0.8660254040\,i\,, \\
x_{4,5} =& -\frac{1}{12}(108 + 12\sqrt{69})^{1/3} - \frac{1}{(108 + 12\sqrt{69})^{1/3}} \pm i\,\frac{\sqrt{3}}{2} \quad \frac{(108 + 12\sqrt{69})^{1/3}}{6} - \frac{2}{(108 + 12\sqrt{69})^{1/3}} \biggr) \\
& \approx -0.662358979 \pm 0.5622795125\,i
\end{aligned}$$

$$\boldsymbol{p_{16}(x) = x^5 - 5x^4 + 1\,0\,x^3 - 10x^2 + 6\,x - 1}$$

$$
\begin{aligned}
x_1 =& -\frac{1}{6}(100 + 12\sqrt{69})^{1/3} - \frac{2}{3(100 + 12\sqrt{69})^{1/3}} + \frac{4}{3} \approx 0.245122333\,, \\
x_{2,3} =& \frac{1}{2}(1 \pm i\,\sqrt{3}) \approx 0.5 \pm 0.8660254040\,i\,, \\
x_{4,5} =& \frac{1}{12}(100 + 12\sqrt{69})^{1/3} + \frac{1}{3(100 + 12\sqrt{69})^{1/3}} + \frac{4}{3} \\
& \pm i\,\frac{\sqrt{3}}{6} \quad \left. \frac{(100 + 12\sqrt{69})^{1/3}}{2} - \frac{2}{(100 + 12\sqrt{69})^{1/3}} \right) \approx 1.877438833 \pm 0.7448617670\,i
\end{aligned}
$$

$$\boldsymbol{p_{17}(x) = x^5 - 6x^4 + 1\,0\,x^3 - 10x^2 + 5\,x - 1}$$

$$
\begin{aligned}
x_1 =& \frac{1}{6}(388 + 12\sqrt{69})^{1/3} + \frac{26}{3(388 + 12\sqrt{69})^{1/3}} + \frac{5}{3} \approx 4.079595625\,, \\
x_{2,3} =& \frac{1}{2}(1 \pm i\,\sqrt{3}) \approx 0.5 \pm 0.8660254040\,i\,, \\
x_{4,5} =& -\frac{1}{12}(388 + 12\sqrt{69})^{1/3} - \frac{13}{3(388 + 12\sqrt{69})^{1/3}} + \frac{5}{3} \\
& \pm i\,\frac{\sqrt{3}}{2} \quad \left. \frac{(388 + 12\sqrt{69})^{1/3}}{6} - \frac{26}{3(388 + 12\sqrt{69})^{1/3}} \right) \approx 0.460202189 \pm 0.1825822549\,i
\end{aligned}
$$

$$\boldsymbol{p_{18}(x) = x^5 + 2\,x^3 + x - 1}$$

$$
\begin{aligned}
x_1 =& \frac{1}{6}(44 + 12\sqrt{69})^{1/3} - \frac{10}{3(44 + 12\sqrt{69})^{1/3}} + \frac{1}{3} \approx 0.5698402912\,, \\
x_{2,3} =& \frac{1}{2}(-1 \pm i\,\sqrt{3}) \approx -0.5 \pm 0.8660254040\,i\,, \\
x_{4,5} =& -\frac{1}{12}(44 + 12\sqrt{69})^{1/3} + \frac{5}{3(44 + 12\sqrt{69})^{1/3}} + \frac{1}{3} \\
& \pm i\,\frac{\sqrt{3}}{2} \quad \left. \frac{(44 + 12\sqrt{69})^{1/3}}{6} + \frac{10}{3(44 + 12\sqrt{69})^{1/3}} \right) \approx 0.2150798545 \pm 1.307141279\,i
\end{aligned}
$$

$$p_{19}(x) = x^5 - x^4 - 2x^2 - 1$$

$$\begin{aligned}
x_1 =& \frac{1}{6}(100 + 12\sqrt{69})^{1/3} + \frac{2}{3(100 + 12\sqrt{69})^{1/3}} + \frac{2}{3} \approx 1.754877667\,, \\
x_{2,3} =& \frac{1}{2}(-1 \pm i\,\sqrt{3}) \approx -0.5 \pm 0.8660254040\,i\,, \\
x_{4,5} =& -\frac{1}{12}(100 + 12\sqrt{69})^{1/3} - \frac{1}{3(100 + 12\sqrt{69})^{1/3}} + \frac{2}{3} \\
& \pm i\,\frac{\sqrt{3}}{2} \quad \left. \frac{(100 + 12\sqrt{69})^{1/3}}{6} - \frac{2}{3(100 + 12\sqrt{69})^{1/3}} \right) \approx 0.1225611669 \pm 0.7448617670\,i
\end{aligned}$$
